# Sustainable hybrid technologies for diclofenac disposal: from innovation to commercial feasibility

**DOI:** 10.1039/d6ra05914g

**Published:** 2026-09-11

**Authors:** Rodrigo Gutiérrez-Ramírez, Samira El Omari, Mehdi Mennani, Leila Azaryouh, Mohamed Laabd, Zineb Kassab, Fabián Fernández-Luqueño, Youness Abdellaoui

**Affiliations:** a Cinvestav Saltillo, Sustainability of Natural Resources and Energy Av. Industria Metalúrgica 1062, Parque Industrial Ramos Arizpe. Ramos Arizpe Coahuila C.P. 25900 Mexico youness@cinvestav.mx; b Laboratory of Materials and Environment, Faculty of Sciences, Ibn Zohr University Agadir Morocco; c Eco-Process, Optimization and Decision Support, Jules Verne University of Picardie (EPROAD, UR4669) F-02100 Saint-Quentin France; d College of Chemical Sciences and Engineering (CCSE), Materials Science, Energy, and Nano-Engineering (MSN) Department, Mohammed VI Polytechnic University (UM6P) Lot 660, Hay Moulay Rachid Benguerir 43150 Morocco; e Equipe des Procédés Chimiques et Matériaux Appliqués (EPCMA), Faculté Polydisciplinaire de Béni-Mellal, Université Sultan Moulay Slimane BP 592 23000 Béni-Mellal Morocco; f Cinvestav Zacatenco, Biotechnology and Bioengineering Department, Av. Instituto Politécnico Nacional 2508 San Pedro Zacatenco Gustavo A. Madero Ciudad de México, CDMX 07360 Mexico; g SECIHTI-Cinvestav Saltillo. Environmental Functional Materials Laboratory, Sustainability of Natural Resources and Energy Ramos Arizpe Coahuila Mexico

## Abstract

The widespread presence of diclofenac (DCF) in wastewater poses significant environmental and health risks, requiring advanced treatment strategies that go beyond conventional methods. This review explores the innovation, scalability, and commercial feasibility of hybrid wastewater treatment systems for efficient DCF removal, highlighting how different treatment processes can be combined and configured according to wastewater characteristics and treatment objectives. Hybrid approaches integrating adsorption, membrane filtration, advanced oxidation processes (AOPs), and biological treatments have demonstrated superior removal efficiencies, often exceeding 90%, due to their synergistic effects. However, their transition from laboratory-scale success to full-scale implementation remains limited by high energy demands, operational costs, and regulatory compliance challenges. This review provides a critical assessment of process optimization, and sustainable engineering strategies that enhance hybrid system performance. Special emphasis is placed on life cycle assessments (LCA) and industrial benchmarks to evaluate the cost-effectiveness and environmental sustainability of emerging technologies, which provide promising pathways toward energy-efficient and scalable wastewater treatment solutions. Additionally, the integration of circular economy principles, including adsorbent regeneration and resource recovery, presents new opportunities for sustainable wastewater management. By bridging technological advancements with commercial viability, this review highlights the potential of hybrid systems to meet real-world industrial and regulatory requirements. The findings offer valuable insights for researchers, policymakers, and the wastewater treatment industry seeking to implement cost-effective, sustainable, and adaptive hybrid technologies for pharmaceutical contaminant removal.

## Abstract 2

The widespread presence of diclofenac (DCF) in wastewater poses significant environmental and health risks, requiring advanced treatment strategies that go beyond conventional methods.

## Graphical abstract

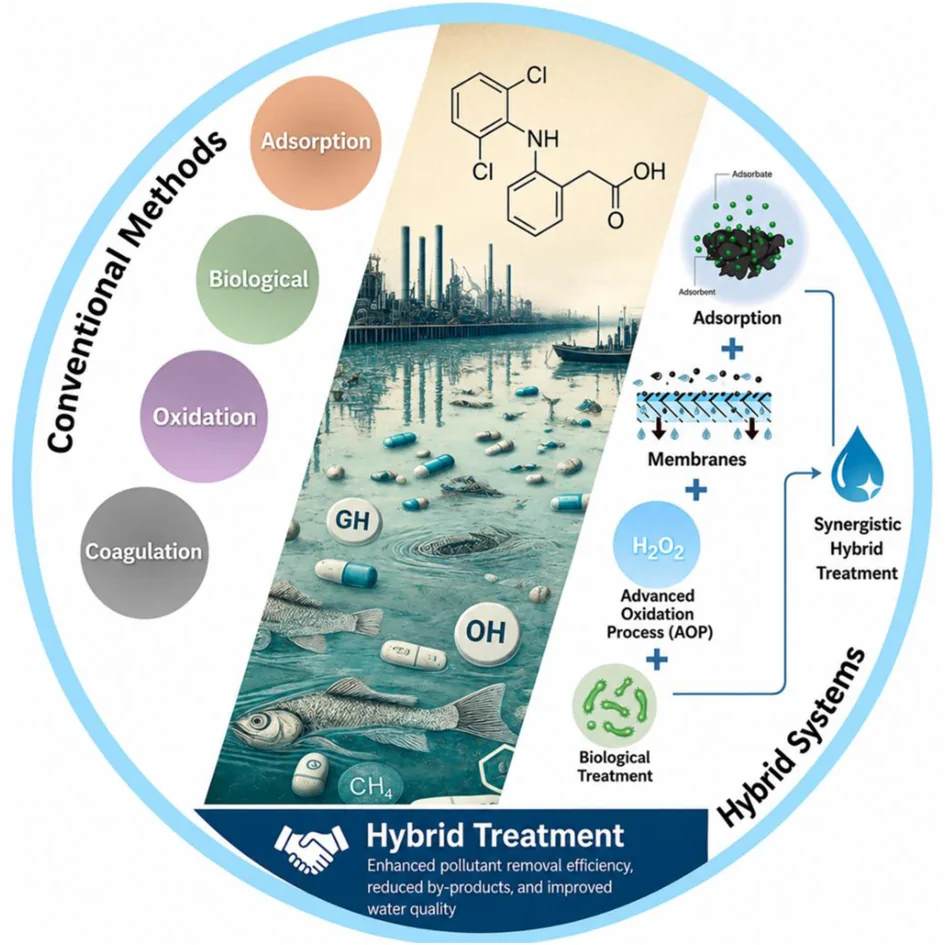

## 1. Introduction

Water is a vital resource essential for sustaining life, with significant socioeconomic and ecological value.^1^ Nowadays, human consumption of this essential resource has become unsustainable. For instance, human activities generate large volumes of wastewater that require proper treatment to prevent the contamination of natural water sources. Emerging contaminants (*e.g.*, pharmaceuticals, pesticides, hormones, personal care products, flame retardants, endocrine-disrupting substances, surfactants and disinfection agents) have recently emerged as a prominent research area for ecologists. These compounds are characterized by limited risk assessment data and the absence of thoroughly detailed regulatory guidelines.^2^ These pollutants are extensively dispersed and can enter aquatic ecosystems *via* multiple wastewater pathways such as healthcare discharges and effluents from wastewater remediation stations.^2,3^ Several studies have reported the presence of these contaminants at different concentrations in polluted waters.^4,5^ Other studies report their detrimental effects, including endocrine disruption, antibiotic resistance, reproductive abnormalities, carcinogenicity, and aquatic toxicity.^6,7^ Nevertheless, they are not regulated by the United States Environmental Protection Agency (US EPA) or by other regulatory bodies worldwide.^8^

DCF is a widespread non-steroidal anti-inflammatory drug available in both oral capsules and as a topical gel.^9^ Despite its therapeutic benefits, DCF has emerged as one of the most concerning micro-pollutants in the environment due to its persistence, toxicity, and widespread occurrence in aquatic ecosystems. Consequently, it has been identified as a priority pharmaceutical pollutant for ecological risk monitoring in the aquatic environment of China and several European countries.^10,11^ DCF has been detected in various aquatic environments, including surface waters at concentrations up to 8000 ng L^−1^,^12^ groundwater at 130 ng L^−1^,^13^ and even drinking water at 4 ng L^−1^,^14^ mainly due to its extensive use to treat different inflammatory disorders. Chronic exposure to DCF has been shown to adversely affect the health of living organisms, causing gastrointestinal damage as well as neurological, cardiovascular, hepatic, renal, hematological, genetic, and developmental toxicities in mammals, thereby affecting ecological balance.^11^ Furthermore, it exhibits semi-persistence in ecosystems, spreading through water and soil and accumulating in the food chains, which could generate potential issues related to its toxicological effects.^15^

DCF removal in wastewater treatment plants is a difficult and ineffective issue.^16,17^ The conventional methods currently used have several shortcomings, including high costs, little operational versatility, and ecological risks associated with DCF intermediate metabolites, which restrict their application in various areas of environmental remediation.^18^ On the other hand, DCF is difficult to eliminate due to the low concentrations (µg L^−1^ or ng L^−1^) in which it is found and its marked polarity.^19^ Moreover, the production of intermediate metabolites throughout the decontamination process of DCF warrants further investigation because of their toxicity.^20^ The presence of DCF and its metabolites in aquatic resource systems may induce the activation of genes conferring antimicrobial resistance, potentially leading to the emergence of antibiotic-resistant bacteria, commonly referred to as superbugs.^21,22^

A significant number of studies have reported the remediation of DCF-containing effluents. Gorito *et al.*,^23^ used biological systems to remove a mixture of micropollutants present in freshwater aquaculture effluents. Sathishkumar *et al.*,^24^ studied the presence of DCF and its metabolites in various environmental compartments, including water sources (surface water, groundwater, drinking water, seawater, and wastewater), soil (soil, sediment, suspended sewage sludge, and leachate), as well as in biota (plants and animals). Vieno and Sillanpää^25^ reviewed the fate of DCF within the human body and throughout the municipal wastewater treatment plant. Other investigations have assessed the effectiveness of advanced decontamination processes (*e.g.*, ozonation, activated carbon) to remove micropollutants in wastewater treatment plants, which might be regarded as the most efficient processes.^26^ Alessandretti *et al.*,^27^ examined the efficacy of various wastewater purification processes in eliminating DCF from water, focusing specifically on the tertiary treatment techniques (*e.g.*, adsorption, membrane separation, and advanced oxidation). Wojcieszynska *et al.*,^28^ analyzed the biodegradation of DCF using immobilized laccases and microorganisms; furthermore, they assessed the toxicity of DCF and its degradation products on non-target organisms.

While individual treatment methods have been extensively studied, previous research has primarily focused on evaluating each technology independently. Consequently, limited attention has been paid to the systematic comparison of hybrid treatment configurations from the perspective of the complementary role of each unit process and the mechanistic synergies responsible for enhancing DCF removal. Understanding these synergies is essential for developing more effective DCF removal strategies, as hybrid approaches may overcome the limitations of single treatment methods through complementary removal mechanisms.^29^ Hybrid wastewater treatment processes are those where two or more conventional techniques (physical, biological, and chemical) are implemented in a sequential order or incorporated into a single system. Hybrid wastewater treatment systems have been widely studied, combining the benefits of existing water treatment technologies to address the limitations and challenges associated with emerging contaminants removal. As an example of their synergistic interaction, they significantly reduced the discharge of emerging contaminants from wastewater treatment plant effluents into receiving ecosystems.^30,31^ Although several previous studies have discussed the application of hybrid process for DCF removal,^27,31,32^ by reporting removal efficiencies or describing individual technologies, this study aims to fill the gap by providing a critical comparison of different hybrid configurations, elucidating the mechanistic role of each treatment stage, evaluating synergistic interactions, assessing the fate of DCF transformation and degradation products, and analyzing operational aspects such as scalability, economic feasibility, and life-cycle considerations. A comparison between the scope of previous reviews and the present work is presented in Table 1. Throughout the review, each hybrid approach is analyzed in terms of efficiency, operational constraints and potential for large-scale implementation, considering the life-cycle impact. This could improve the sustainability and profitability of hybrid systems. Finally, future research directions are described to support the development of intelligent, scalable and commercially viable wastewater treatment technologies for DCF and other emerging contaminants.

**Table 1:** Comparison of the scope and key aspects addressed in previous reviews and the present review on hybrid treatment systems for diclofenac removal^a^

Reference	DCF	Individual technologies	Hybrid configurations	Mechanistic synergy	Transformation products	Scalability/techno-economic	LCA	Future perspectives
Alessandretti *et al.* (2021)	✓	✓	L	✗	L	✓	✓	✓
Eniola *et al.* (2022)	L	✓	✓	L	L	✓	✓	✓
Ponnusami *et al.* (2023)	✗	L	L	✓	L	✓	✓	✓
This review	✓	✓	✓	✓	✓	✓	✓	✓

a L = Limited.

## 2. Diclofenac – properties, potential and impacts

DCF is a phenylacetic acid derivative (C_14_H_11_Cl_2_NO_2_, 296.15 g mol^−1^).^11^ Its structural configuration, featuring two chlorine atoms, results in moderate lipophilicity (log *P* = 4.51) and aqueous solubility at neutral pH of 50 mg mL^−1^ at 25 °C. With a p*K*_a_ of 4.15, DCF exists predominantly in anionic form under alkaline conditions, while precipitating in acidic environments.^11,33^ DCF is available in two salt forms: sodium and potassium. Table S1 presents a summary of the main physicochemical properties of DCF sodium and DCF potassium. These characteristics are key factors governing the environmental behavior and removal of DCF, as they influence its ionization state, solubility, and affinity for adsorbing surfaces. The sodium salt, which is slowly absorbed in the duodenum, is more suitable for managing chronic pain, whereas the potassium salt, rapidly absorbed in the stomach, is intended for the treatment of acute pain. The high log *P* value and dipole moment (2.51 D) indicate significant bioaccumulation potential in biological tissues.^27,34^

Global DCF consumption reaches approximately 2400 tons annually, with applications in both human and veterinary medicine. The market is projected to grow at 3.87% annually, reaching $5.64 billion by 2025.^11^ However, its widespread use has raised environmental concerns. Notable impacts include the catastrophic decline (97–99.9%) of Gyps vulture populations in South Asia and toxic effects on aquatic organisms at concentrations up to 6.27 mg L^−1^.^35,36^ These concerns have led to DCF's classification as an emerging contaminant and its inclusion in the EU Water Framework Directive Watch List. The compound's environmental persistence and bioaccumulation potential threaten both ecological systems and human health, prompting global regulatory responses, including bans in several Asian countries and EU monitoring programs.^37,38^ This situation necessitates advanced monitoring and elimination technologies in wastewater treatment to protect environmental and public health.

## 3. Conventional methods for DCF removal

Conventional wastewater treatment methods for DCF removal include adsorption, membrane filtration, biological treatment and AOPs. Although some of these methods, in particular activated carbon adsorption and membrane filtration, have been adopted in large-scale wastewater treatment plants, they are often insufficient as stand-alone solutions. Their limitations in treating low concentrations, high operational costs, and regulatory constraints mean the use of hybrid treatment approaches, which combine several techniques, to improve efficiency and sustainability.

Adsorption facilitates the transfer of substances from liquid or gas phases onto solid materials through physicochemical interactions, offering advantages of operational simplicity and cost-effectiveness. While activated carbon remains highly effective due to its porosity and large surface area (over 1000 m^2^ g^−1^), its high production costs have driven research toward alternative adsorbents derived from natural sources like leather waste, rice hulls, and clays.^27,39^ The process efficiency depends on both adsorbate characteristics (electrical charge, molecular structure, solubility) and adsorbent parameters (active sites, surface area, pore size), involving mechanisms such as electrostatic interaction, ion exchange, and hydrogen bonding.^31^ Table S2 summarizes recent research on DCF adsorption using various synthesized adsorbents (*e.g.*, clays, carbon nanotubes, chitosan, molecularly imprinted polymer, hydrogels, *etc.*). The results showed that conventional clays exhibit moderate adsorption capacities (23.83–238 mg g^−1^), while nanostructured materials offer significantly superior performance, notably with an exceptional uptake capacity of 892.85 mg g^−1^ for L@M-MWCNT. Based on the p*K*_a_ value (4.15) of DCF, pH significantly influences adsorption efficiency. Acidic conditions (pH < p*K*_a_) promote non-electrostatic interactions such as hydrogen bonding and π–π stacking. Conversely, at pH > p*K*_a_, the adsorption is controlled by electrostatic interactions between the DCF and negatively charged adsorbent surface.^27^ Therefore, DCF adsorption is primarily governed by multiple mechanisms, namely hydrogen bonding, electrostatic interactions, and π–π interactions. The possible adsorption mechanisms of DCF are illustrated in Fig. S1.

Membrane filtration is one of the most consistently effective tertiary water treatments, operating as a selective filter between distinct phases. The separation process is controlled by driving force, component mobility, and concentration gradients at the interface.^40^ These systems have been recognized as powerful processes in water purification owing to their cost-effectiveness, scalable production capacity, and efficient micropollutant removal. In general, ultrafiltration (UF) and microfiltration (MF) are generally ineffective at removing micropollutants, because their pore diameters are bigger than micropollutants' molecular size.^41^ Nanofiltration (NF) and reverse osmosis (RO) are the most effective membrane processes for removing DCF, primarily through size exclusion and electrostatic repulsion. Licona *et al.*^42^ evaluated the performance of NF membrane (NF 90) and RO membrane (BW30) for the removal of 10 mg L^−1^ of DCF. The rejection for both membranes was up to 90% and 98%, respectively, under experimental conditions (pH 5 and 20 bar), and was attributed to the competition between the steric and electrostatic mechanisms. Table S3 compares various nanofiltration (NF) and reverse osmosis (RO) systems in terms of separation performance for DCF removal. The data reveal that these two filtration methods offer remarkably high yields, often exceeding 90%, with a maximum performance of 99.5–99.7% achieved by PIP-KRO, RO4, and NF50 membranes. In contrast, the poly(vinyl alcohol)-based membrane modified with citric acid, glycerol, and silver nanoparticles synthesized using a green synthesis method exhibits a relatively lower DCF removal efficiency (80%). These findings also show that performance depends not only on the membrane process (NF or RO) but also on the membrane's structure and design. Key factors influencing membrane performance include pore size and membrane type,^43,44^ and operational conditions, as well as practical challenges such as membrane fouling, long-term system stability and pollutant degradation.^45^ Moreover, high installation and maintenance costs, as well as the issue of concentrated residue accumulation in the membranes, limit their large-scale application.

Biological wastewater treatment encompasses aerobic and anaerobic processes, including activated sludge, constructed wetlands, membrane bioreactors (MBR), and enzyme treatments. The biodegradation process, where microorganisms break down pollutants into less toxic forms, offers economic benefits under moderate operational conditions.^46^ Several degradation mechanisms participate in the biological treatment of DCF, including hydroxylation of benzene rings, amidation/de-amidation, sulfate conjugation of phenol functions, reductive dechlorination, oxidative ring-opening, and oxidation by dehydrogenation of phenol functions.^47^ However, effectiveness depends on factors like molecular composition, drug concentration, and operational parameters (*e.g.*, pH, temperature and light), with limitations for non-biodegradable contaminants. For DCF biodegradation, while microbial degradation presents an environmentally friendly approach, the compound can damage cell membranes and disrupt bacterial functionality.^48^ The performance of various biological treatment processes for the removal of DCF is reported in Table S4. The data revealed that the choice of biological system strongly influences DCF removal efficiency. For instance, certain bacterial strains (*Bacillus subtilis* and *Brevibacillus laterosporus*) and horseradish peroxidase immobilized on perlite achieved maximum DCF removal efficiencies of 100%.^49,50^ Besides, the enhanced biological phosphorus removal (EBPR) process,^51^ and high-concentration aerobic activated sludge systems^52^ can achieve high DCF degradation efficiencies (>98%). In addition, an assembled mixed co-culture (*P. oxalicum* XD-3.1, *P. rastrickii*, and *C. cladosporoides*) achieved more than 80% DCF degradation with a 90% reduction in toxicity. In contrast, the biodegradation systems based on microalgae, indigenous microflora, or immobilized laccase achieve intermediate DCF removal efficiencies (70–90%). Meanwhile, bio-electrochemical systems (BES) based on electro-active microorganisms and electrodes exhibit low to moderate DCF removal yields. Nevertheless, DCF may adversely affect the microbial community and overall biological treatment performance. Zhao *et al.*^53^ reported that DCF inhibited anaerobic phosphorus release and aerobic phosphorus uptake in EBPR systems. At a DCF concentration of 2.0 mg L^−1^, the removal efficiencies of COD, NH_4_^+^-N, and soluble orthophosphate decreased significantly, demonstrating that DCF can interfere with microbial metabolic activity and compromise biological treatment performance.

Advanced oxidation encompasses diverse technologies including photocatalytic oxidation, Fenton and Fenton-type oxidation, photolysis, ozonation, electrochemical oxidation, and sulfate radical-based advanced oxidation process (AOP-SR). Fig. S2 presents a schematic overview of the main AOPs (*e.g.*, ozonation, electrochemical oxidation, Fenton oxidation, photolysis, photocatalysis and sonolysis), which highlights the diversity of oxidation strategies currently available for degrading DCF in wastewater. Because DCF is photosensitive, its main decomposition in surface waters occurs *via* light-induced photolysis.^54^ This process generates carbazole compounds, which are believed to drive the phototoxic effects of the DCF intermediate products.^55^ Advanced oxidation processes generate reactive oxygen species (hydroxyl radicals, superoxide radicals, and sulfates) that convert complex pollutants into simpler products like H_2_O and CO_2_. Efficiency depends on operating conditions, reactive oxygen species production, and mass transfer resistance.^32,56^ Plasma generation through electrical discharges represents another advanced oxidation approach, encompassing pulsed corona discharge, atmospheric cold plasma discharge, and dielectric barrier plasma. In pulsed corona discharge, an electric field applied between water-submerged electrodes creates discharges that ionize gases, producing reactive radicals (OH, O_3_, N_2_, and HO_2_) through high-energy electron dissociation.^57^ Dobrin *et al.*,^58^ achieved complete DCF removal (50 mg L^−1^) within 15 minutes, with 50% TOC mineralization after 30 minutes and an energy yield of 0.76 g kW^−1^h^−1^ for 90% conversion. Banaschik *et al.*,^59^ degraded DCF (500 µg L^−1^) using a coaxial geometry reactor with 80 000 discharges, achieving an energy yield of 45 mg kW^−1^h^−1^ and requiring 27 kWh m^−3^ to attain 90% DCF removal.

## 4. Hybrid methods for DCF removal and degradation

Tertiary techniques have been employed for the removal and degradation of emerging contaminants present in polluted water effluents.^60,61^ However, the efficiency of these techniques is highly related to the nature of the contaminant to be removed. The variety of compounds present in the effluent and their properties, such as molecular weight, charge, aromaticity, hydrophobicity and functional groups, influence the removal effectiveness and the reduction of emerging contaminants to the required levels. For this reason, intensive tertiary treatment processes, including the integration of two or more treatment operations, have emerged with the aim of: (i) treating and reusing various contaminated water effluents; (ii) increasing the efficiency of emerging contaminant removal; and (iii) reducing the operational costs and carbon footprint of water treatment plants.^27^ The integration of adsorption, AOPs, membrane filtration, biological systems, and other treatment technologies into hybrid is discussed throughout this section of the text, considering studies focused on the effective removal and degradation of DCF. Although these integrated systems often involve higher initial investment and energy demands, they provide enhanced long-term sustainability through improved treatment efficiency, greater reduction of pollutant loads, and the potential for safe water reuse.^62^Table 2 summarizes the specific role of each unit process in representative hybrid treatment systems and highlights how their combination contributes to the overall performance of diclofenac removal. The accompanying discussion in the following section has also been expanded to clarify the synergistic interactions between the different treatment stages.

**Table 2:** Mechanistic roles of individual unit processes and their synergistic interactions in representative hybrid treatment configurations for diclofenac removal

Hybrid configuration	Role of first unit process	Role of second unit process	Mechanistic synergy
Adsorption-biological	Rapidly removes DCF and other inhibitory micropollutants, buffering concentration peaks	Biodegrades remaining biodegradable organics and residual DCF/intermediates	Lower DCF concentrations reduce toxic stress on microorganisms, preserving microbial activity and enhancing biodegradation efficiency
Biological-adsorption	Removes biodegradable organic matter (BOD/COD) and part of the pharmaceutical load	Polishes the effluent by absorbing persistent DCF and other recalcitrant micropollutants	Lower organic matter reduces competition for adsorption sites and fouling, increasing adsorbent capacity and extending its service life
AOPs – biological	Oxidizes DCF into smaller, more oxygenated intermediates through hydroxyl radicals or other reactive species	Mineralizes oxidation by-products that become more biodegradable	Oxidative transformation increases biodegradability (higher BOD/COD ratio), allowing microorganisms to more efficiently degrade the oxidation products
Biological – AOPs	Removes readily biodegradable compounds, decreasing the bulk organic load	Oxidizes residual DCF and refractory transformation products	Lower organic load reduces radical scavenging and oxidant consumption, improving AOP efficiency and reducing energy/chemical demand
Adsorption-membrane	Removes dissolved DCF and part of the dissolved organic matter before membrane separation	Provides physical separation of remaining contaminants and suspended solids	Lower organic load decreases membrane fouling and concentration polarization, maintaining permeate flux and extending membrane lifespan
Membrane – adsorption	Retains suspended solids, colloids and macromolecules	Adsorbs dissolved DCF escaping membrane retention	Pretreatment minimizes pore blockage of the adsorbent and improves mass transfer, enabling more efficient adsorption of low-molecular-weight contaminants
AOPs-adsorption	Partially oxidizes DCF and natural organic matter, reducing molecular complexity	Captures residual DCF and oxidation by-products	Partial oxidation exposes new functional groups and decreases contaminant load, reducing competition for adsorption sites and improving polishing efficiency
Adsorption-AOPs	Removes a significant fraction of DCF and competing organic compounds	Oxidizes remaining DCF and adsorbed desorption products to achieve complete removal	Lower contaminant concentration decreases oxidant demand and energy consumption while minimizing the formation of undesirable oxidation by-products
AOP-membrane	Reduces the DCF concentration through oxidation and transforms it into smaller TPs	Retain residual DCF, TPs, and other organic species, and provide an effluent polishing stage	Pre-oxidation reduces DCF and organic foulant concentrations, mitigating membrane fouling while enhancing the removal of residual DCF and TPs
Membrane-AOPs	Selective separation of organic contaminants by pore size exclusion, electrostatic interactions. Concentration of DCF.	Oxidative degradation of residual or concentrated DCF and TPs	Pretreatment reduces the contaminant load and concentrates DCF in the retentate, facilitating subsequent oxidation while potentially reducing oxidant demand and energy consumption

### 4.1. Adsorption – AOPs

The combination of AOPs and adsorption aims to create synergy between them, leveraging the advantages while minimizing the drawbacks of each operation. The integration of AOPs and adsorption processes has been applied by alternating the sequence between them, either adsorption followed by the AOPs stage or *vice versa* (Fig. 1). The integration mode of these technologies could lead to variations in terms of treatment efficiency, operation time, generated residues, operational costs, and other factors.

**Fig. 1:**
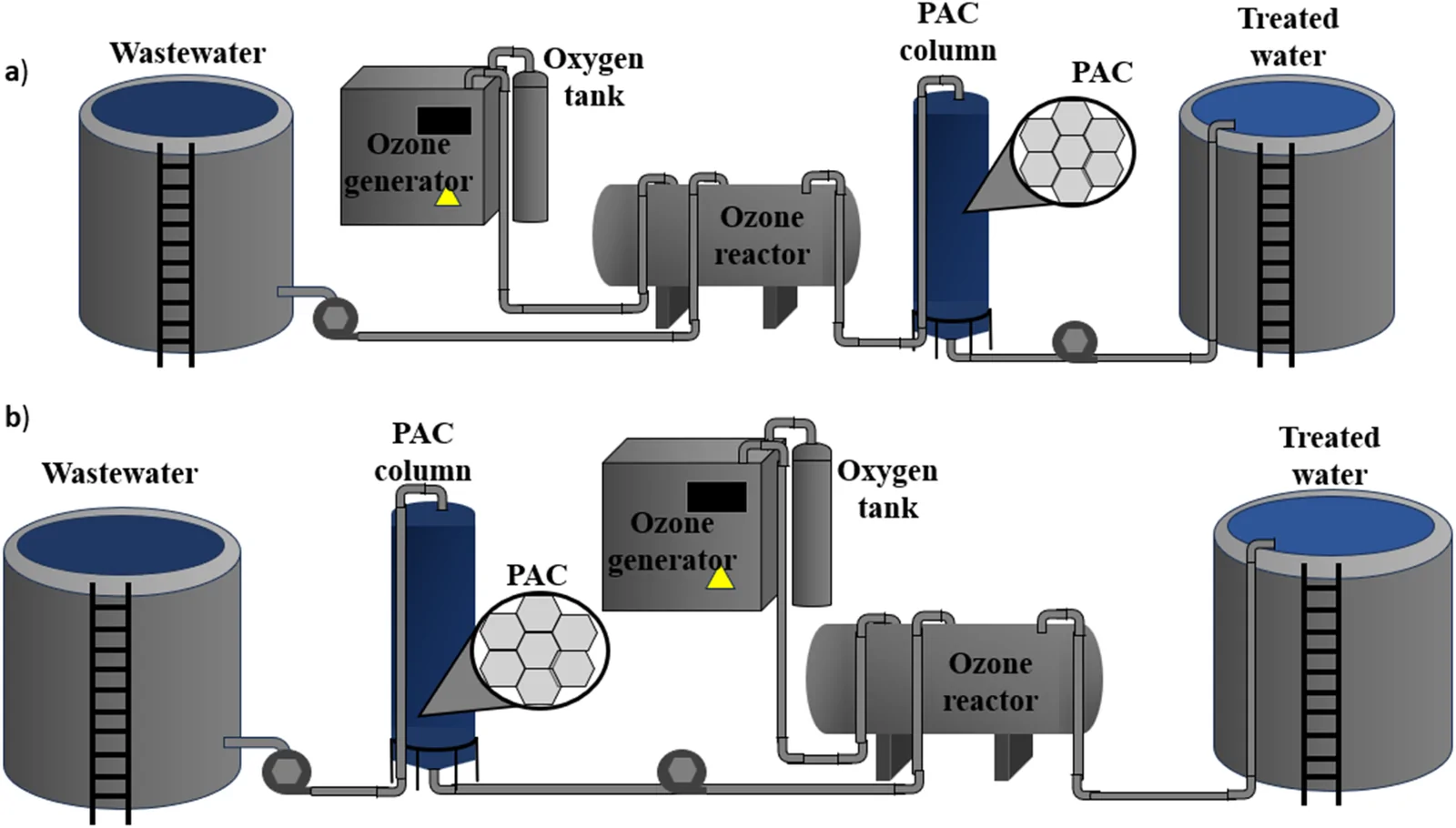
Schematic representation of the two hybrid ozonation–adsorption configurations: (a) ozonation followed by adsorption in a powdered activated carbon (PAC) column and (b) adsorption in a PAC column followed by ozonation.

The first implementation mode involves the sequential combination of chemical degradation by AOPs follow by adsorption processes (Fig. 1a). In this approach, AOPs are commonly applied as a pretreatment step to partially degrade target pollutants and other organic constituents present in the effluent through the action of highly reactive species such as hydroxyl radicals. This pretreatment reduces the concentration of competing compounds and transforms contaminants into more easily removable species, thereby improving the subsequent adsorption performance and decreasing adsorbent consumption.^63^ Adsorption then acts as a polishing step, removing residual pollutants and oxidation by-products that may remain after the oxidation treatment. Several studies have demonstrated the effectiveness of this synergistic approach for DCF removal. As an example, Esquerdo *et al.*,^64^ reported that the sequential O_3_/PAC system significantly improved DCF removal efficiency while minimizing the formation of oxidation by-products compared with ozonation alone. Similarly, Rozas *et al.*,^65^ showed that combining ozonation with PAC enhanced toxicity reduction by decreasing the generation of toxic intermediates. These findings demonstrate that coupling AOPs with adsorption can improve pollutant removal efficiency while simultaneously enhancing effluent quality and reducing potential environmental risks associated with oxidation by-products.

It has been reported that adsorbent materials, such as activated carbon, are not only effective in the adsorption of contaminants, but also can act as a reaction support and as promoters in the generation of radicals in ozonation processes.^63^ The activated carbon surface can act as a catalyst, accelerating the conversion of O_3_ into OH free radicals, which are useful for the mineralization of the micropollutants, increasing the overall process efficiency. The mineralization process is related to the decline in total organic carbon (TOC), which is a measure of the total amount of organic compounds in the water. As an example, Beltrán *et al.*^66^ demonstrated that the combination of ozonation with activated carbon accelerated DCF degradation and significantly improved TOC removal compared with adsorption and ozonation alone. Catalytic reactions were the key step for the mineralization of DCF byproducts due to the accumulation of ozone layer in water. The presence of activated carbon promoted the decomposition of ozone into H_2_O_2_ and OH radicals, which improved the removal of TOC (95%) compared to simple ozonation (40%).

The second implementation mode involves the use of adsorption as a pre-treatment step prior to oxidation processes (Fig. 1b). In this approach, adsorption is used to remove organic matter and other competing compounds from the effluent before oxidative treatment. The reduction of matrix complexity and interfering compounds ensures a more effective degradation of target pollutants and minimizes oxidant consumption. Additionally, this pretreatment approach can enhance overall oxidation efficiency and limit the formation of undesirable transformation by-products, resulting in improved treatment performance and effluent quality. In this sense, Brienza *et al.*^67^ implemented a hybrid adsorption-oxidation treatment by combining PAC and clay adsorption with photo-Fenton oxidation for DCF removal. PAC adsorption significantly reduced the organic load, total organic carbon (TOC), while micelle clay enhanced DCF removal due to the strong affinity between the negatively charged DCF molecules and the cationic sites of the clay material. Following adsorption, the residual DCF was efficiently degraded through a sunlight-driven photo-Fenton process using peroxymonosulfate. This approach emphasizes the significance of adsorption as a preliminary treatment to increase water transmittance, which in turn enhances photolysis performance and supports the development of efficient hybrid wastewater treatment systems.

### 4.2. Adsorption – membrane filtration

The combination of multiple treatment technologies within intensive water treatment processes has become a relevant topic in environmental research. Among these approaches, hybrid membrane processes that integrate ultrafiltration with adsorption represent a promising alternative for the removal of emerging pollutants and organic matter from water, improving its quality by combining the advantages of both individual processes. According to Davoodbeygi *et al.*,^68^ membrane-adsorption hybrid systems can be classified into three configurations: pre-adsorption, post-adsorption, and synergistic integration, where both processes are unified in a single process. Huang *et al.*,^69^ demonstrated that the sequence combination of activated carbon adsorption and NF membrane process was more effective in removing DCF than NF-AC combination. The adsorption process as pretreatment reduces the concentration of contaminants or organic compounds in the wastewater, while membrane filtration serves to retain the adsorbent material and remain suspended or larger pollutants (Fig. 2). This configuration can also contribute to control membrane fouling because the pretreatment stage removes dissolved organic matter and other potential foulant before the wastewater reaches the membrane process. In wastewater containing DCF, organic compounds, colloids, suspended solids, among other dissolved organic constituents can accumulate on the membrane surface or enter the membrane pores, promoting cake-layer formation, generating pore blocking, and a progressive increase in transmembrane pressure. By decreasing the concentration of foulants upstream, the adsorption process can limit their deposition on the membrane surface, thereby mitigating membrane fouling and improving effluent quality to enable its reuse in irrigation systems or aquifer recharge.^70^ The synergistic effect can increase the overall pollutant removal efficiency, reduce transmembrane pressure and membrane fouling, extend operational membrane lifetime, and optimize operational costs.^71^

**Fig. 2:**
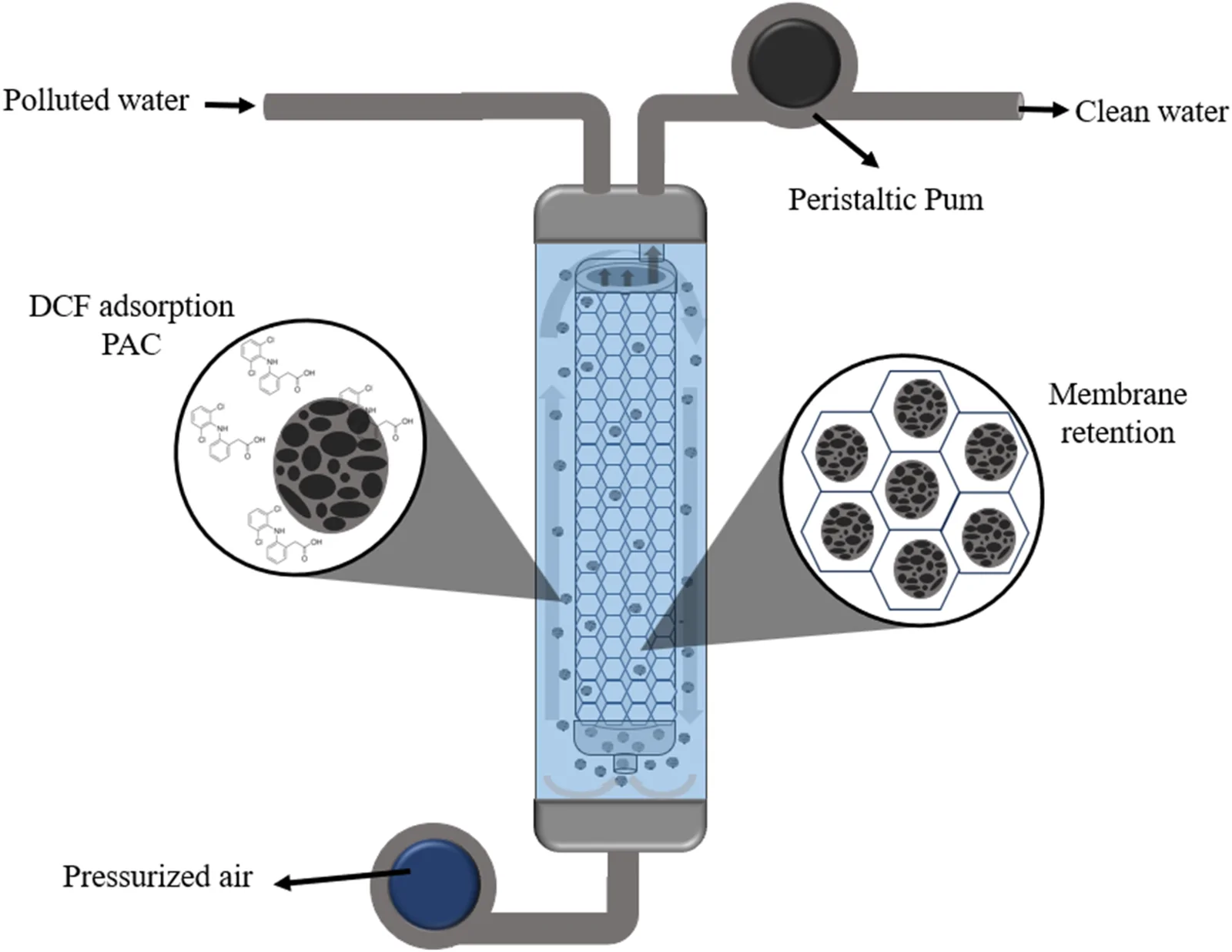
Schematic representation of adsorption-membrane hybrid process.

The efficiency of this hybrid technology depends on several factors, including the quality of the treated water, system configuration, membrane type, adsorbent dosage, the nature of target pollutants, among others.^72^ Additional factors, such as operating conditions like pressure and flow rate, the presence of competing compounds, and membrane fouling, are also important. The dosage of adsorbent is a crucial factor that determines the effectiveness in removing contaminants from water. Therefore, optimizing the amount of adsorbent is essential to guarantee high removal efficiency, avoiding membrane fouling and premature saturation of the material and, at the same time, minimizing the operating costs of the process.^68,73^ Ivančev-Tumbas *et al.*^74^ studied the efficiency of the hybrid process in relation to the effect of PAC dosage and its continuous or single addition. The increase in PAC doses improved the DCF removal until the addition of 15 mg L^−1^. The addition of higher doses of PAC generated PAC packing or improper distribution within the column under the conditions used. Additionally, single PAC dosing (38%) was more effective than continuous dosing (32%) in terms of average cycle removal efficiency. This was because the PAC added at the beginning of the process reaches equilibrium with the initial adsorbate concentration in the effluent, resulting in higher surface loading on the PAC. Finally, they reported that the DCF removal efficiency using PAC/UF ranged from 25% to 41%. They attributed the low yield obtained due to the formation of PAC agglomerates due to the high dose of stock solutions. The formation of agglomerates decreased the capillary effect, affecting the transport of the contaminant. These findings highlight the influence of matrix complexity on adsorption-based hybrid systems and emphasize the importance of optimizing adsorbent dosage according to effluent characteristics to maintain effective contaminant removal.

Adsorption processes with PAC and granular activated carbon (GAC) as pretreatment have been widely used to enhance the operational performance of membranes, as they are effective and efficient in adsorbing dissolved organic carbon (DOC), thus preventing membrane fouling, reducing transmembrane pressure (TMP) and enhancing the productivity and applicability of the membrane.^72^ Löwenberg *et al.*,^75^ removed DCF and other micropollutants, including sulfamethoxazole, carbamazepine, mecoprop and benzotriazole using submerged (sPAC/UF) and pressurized (pPAC/UF) systems, which operated continuously for six months. Both membrane systems remained stable without deterioration or abrasion over the six-month period, demonstrating their feasibility for the post-treatment of secondary wastewater effluents. The hybrid adsorption-ultrafiltration systems effectively reduced dissolved organic carbon (DOC), including biopolymers, humic substances, and low-molecular-weight organic compounds. In these systems, PAC played a major role in the removal of humic substances and smaller organic molecules through adsorption, whereas UF membranes preferentially retained larger biopolymeric fractions. The combination of both mechanisms contributed to improved overall organic matter removal and enhanced DCF elimination. Among the evaluated configurations, the pPAC/UF system (85%) showed slightly higher DCF removal efficiency than the sPAC/UF system (83%). In terms of operational performance, the pPAC/UF system operated at higher flow rates (80 L m^−2^ h^−1^) and showed better permeability. Moreover, the energy demand of the pPAC/UF was lower (0.056 kWh m^−3^) than that of the sPAC/UF (0.178 kWh m^−3^), making it a more energy-efficient alternative for hybrid wastewater treatment systems. These indicate that process configuration influences not only the interaction between adsorption and membrane separation mechanisms, but also in energy demand and operational performance.

### 4.3. AOPs – membrane filtration

The integration of AOPs with membrane technologies has been explored as an effective strategy for enhancing DCF removal from wastewater. In this hybrid system, AOPs generate highly reactive species, particularly hydroxyl radicals (˙OH), that break down complex organic molecules like DCF into smaller and less toxic by-products.^76,77^ However, because some degradation intermediates may persist in the treated effluent, membrane filtration is incorporated as a complementary polishing step to retain by-products or complete DCF removal. The synergistic combination of AOP-membrane improves pollutant removal efficiency, and effluent quality. AOPs as pretreatment step can mitigate membrane fouling by decomposing high molecular weight compounds and recalcitrant organic compounds into smaller molecules before filtration. This reduces the accumulation of organic foulants on the membrane surface, limiting cake-layer formation and pore blocking. Consequently, the reduction in blockages can help to maintain membrane permeability, decrease transmembrane pressure and energy consumption required for operation and maintenance requirements.^78,79^ These systems consistently achieve high removal efficiencies exceeding 90%, producing water of significantly higher quality than conventional treatment methods.^80,81^ Research endeavors have been directed towards optimizing both the standalone and integrated systems to address challenges such as membrane fouling, energy utilization, and the incomplete degradation of pharmaceutical contaminants.^82^

Apopei *et al.*^83^ investigated a hybrid photocatalytic-filtration system based on granular activated carbon-supported TiO_2_ nanoparticles (GAC-TiO_2_N) for DCF removal (Fig. 3A). In this system, the porous structure of GAC promoted DCF adsorption, while TiO_2_ nanoparticles generated reactive oxygen species (ROS) under UV irradiation, enabling the photocatalytic degradation of the adsorbed contaminant. The study evaluated pH conditions (Fig. 3B) and demonstrated that alkaline conditions enhanced the performance of the hybrid material, which was attributed to the increased generation of hydroxyl radicals (˙OH) and the consequent improvement in photocatalytic activity. In addition, SEM images (Fig. 3C and D) revealed that the GAC-TiO_2_N composite preserved its porous structure and overall integrity after the filtration-photocatalytic process, despite minor surface modifications associated with the accumulation of reaction by-products. These findings suggest that the material maintains its adsorption and catalytic properties after repeated treatment cycles, highlighting its potential as a durable and reusable material for hybrid water treatment applications.

**Fig. 3:**
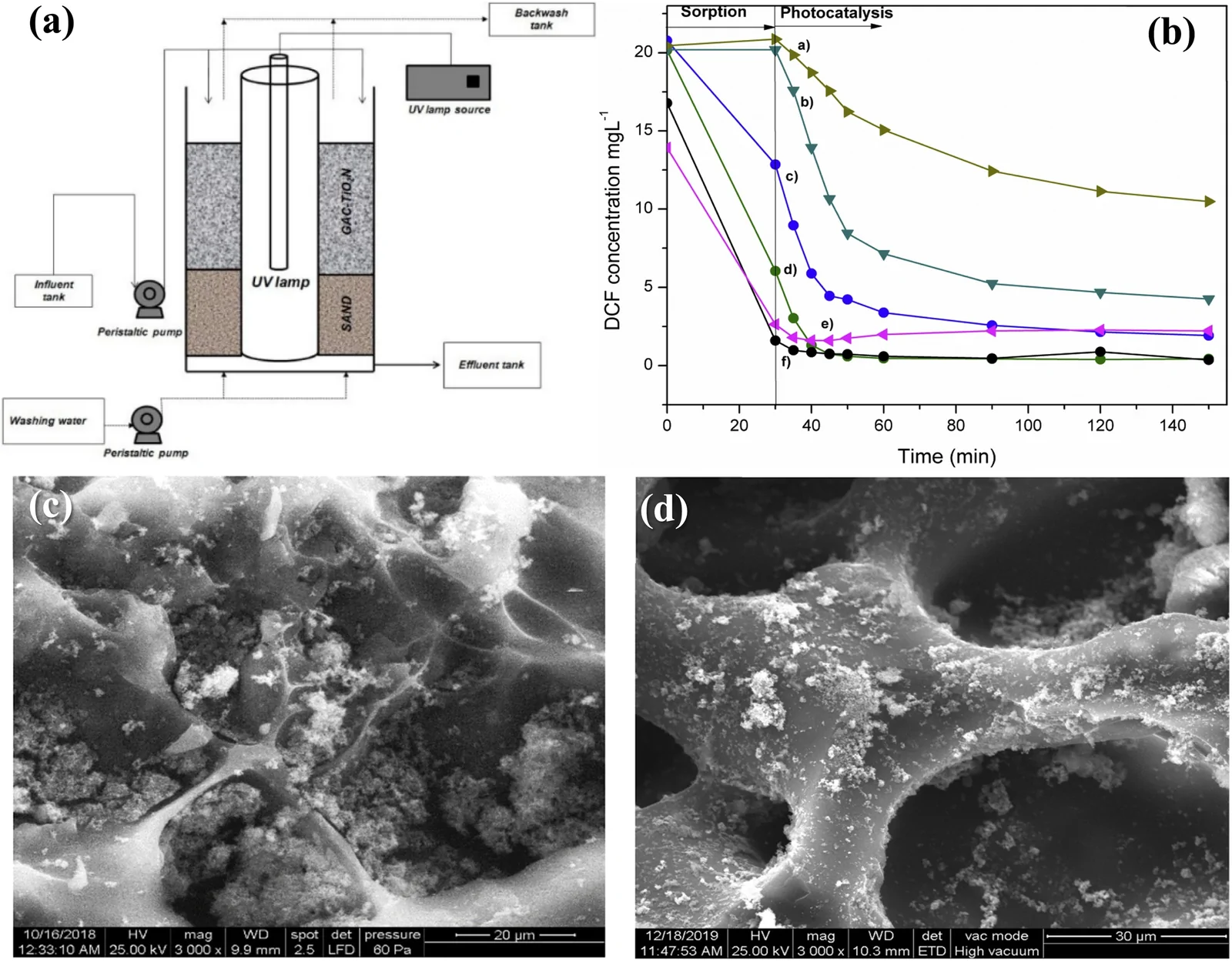
Hybrid AOP-filtration system for DCF removal: (a) fixed-bed column set-up, (b) time-evolution of DCF degradation using GAC-TiO_2_N under different pH conditions, and SEM images of GAC-TiO_2_N before (c) and after (d) photocatalytically assisted filtration, adapted from ref. 83 with permission from Elsevier P. Apopei, C. Orha, M. I. Popescu, C. Lazau, F. Manea, C. Catrinescu and C. Teodosiu, *Process Safety and Environmental Protection*, 2020, 138, 324–336, copyright 2020.

Dekkouche *et al.*,^84^ also explored the integration of membrane filtration and photocatalysis through the development of TiO_2_-modified polysulfone membranes for DCF removal under UV irradiation (Fig. 4a and b). The incorporation of TiO_2_ into the polymeric matrix increased the available reactive surface area, enhancing photocatalytic activity as the water permeated through the membrane. As a result, the hybrid membrane system achieved high DCF removal efficiencies (>90%), demonstrating the potential of photocatalytic membranes as multifunctional materials for simultaneous filtration and degradation of emerging contaminants.

**Fig. 4:**
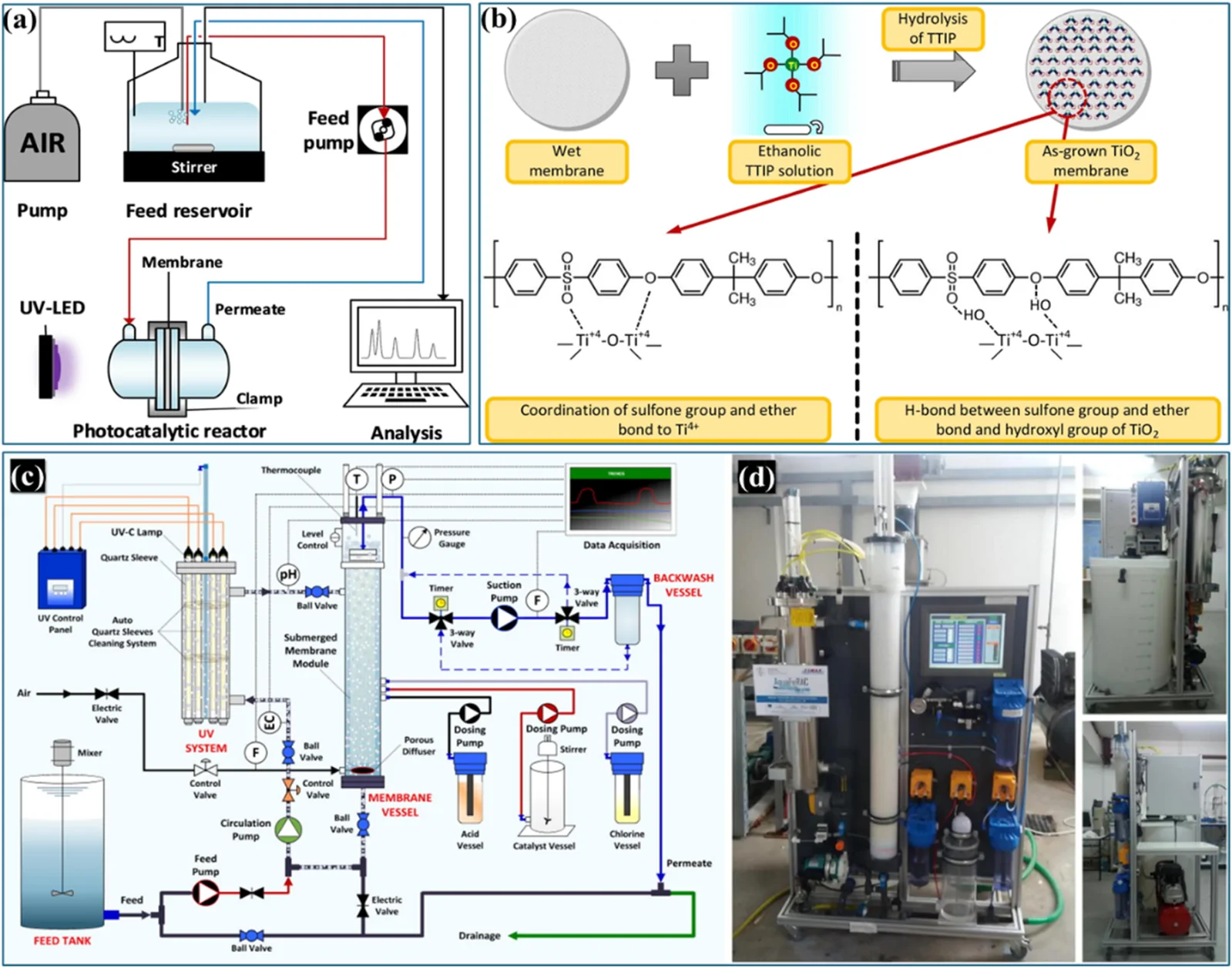
(a) Lab-scale photocatalytic reactor setup with UV-LED activation, (b) Interaction between polysulfone polymer membrane and TiO_2_ during membrane modification, reproduced from ref. 84 with permission from Elsevier Dekkouche, Seghir, Sergio Morales-Torres, Ana R. Ribeiro, Joaquim L. Faria, Clàudia Fontàs, Ounissa Kebiche-Senhadji, and Adrian MT Silva. *Chemical Engineering Journal* 427: 131476, copyright 2022. (c) Pilot-scale continuous photocatalytic membrane reactor system with UV-C light source and backwash mechanism, (d) Front and side views of the pilot-scale reactor system, adapted from ref. 85 with permission from Elsevier K. V. Plakas, V. C. Sarasidis, S. I. Patsios, D. A. Lambropoulou and A. J. Karabelas, *Chemical Engineering Journal*, 2016, 304, 335–343, copyright 2016.

This hybrid process has also been explored at pilot scale to improve the practical applicability of hybrid systems for wastewater treatment, particularly for pharmaceutical contaminants. In this context, Plakas *et al.*^85^ developed a continuous photocatalytic membrane reactor combining suspended TiO_2_ photocatalysis with UF. In this hybrid configuration (Fig. 4c and d), DCF was initially degraded through photocatalytic oxidation, followed by the gradual degradation of its transformation products, while the UF membrane simultaneously retained TiO_2_ particles and prevented catalyst release into the treated effluent. Another promising hybrid strategy involves the integration of electrochemical oxidation with membrane bioreactor technology in electro-membrane bioreactors (EMBRs). Ensano *et al.*^86^ demonstrated the applicability of this approach for DCF removal, achieving significant degradation efficiencies through the synergistic interaction between both treatments. In this configuration, reactive oxygen species (ROS), particularly hydroxyl radicals (˙OH), were generated electrochemically to promote the degradation of pharmaceutical contaminants, while the membrane module retained suspended solids, microorganisms, and residual organic matter. In addition, aeration enhanced oxidation performance by increasing oxygen availability, whereas periodic membrane backwashing help to mitigate membrane fouling and maintain stable operation. These findings highlight the potential of EMBR systems as multifunctional platforms for efficient and continuous wastewater treatment.

Plakas *et al.*^87^ investigated and studied a Fenton-type reaction integrated with a ceramic microfiltration membrane for the removal of DCF. By immobilizing an iron-based catalyst on the membrane, the system reduces the need for continuous iron dosing and minimizes sludge production, a common problem in traditional homogeneous Fenton reactions (Fig. 5a–d). The α-Al_2_O_3_/*n*Fe membrane module serves as both a physical filter and a catalytic surface for the Fenton-type reaction, breaking down DCF into less harmful by-products when H_2_O_2_ is introduced. Using response surface methodology (RSM), the study optimized operating conditions, achieving a DCF removal efficiency of 65% at pH 3 and a H_2_O_2_ concentration of 42.9 mg L^−1^.

**Fig. 5:**
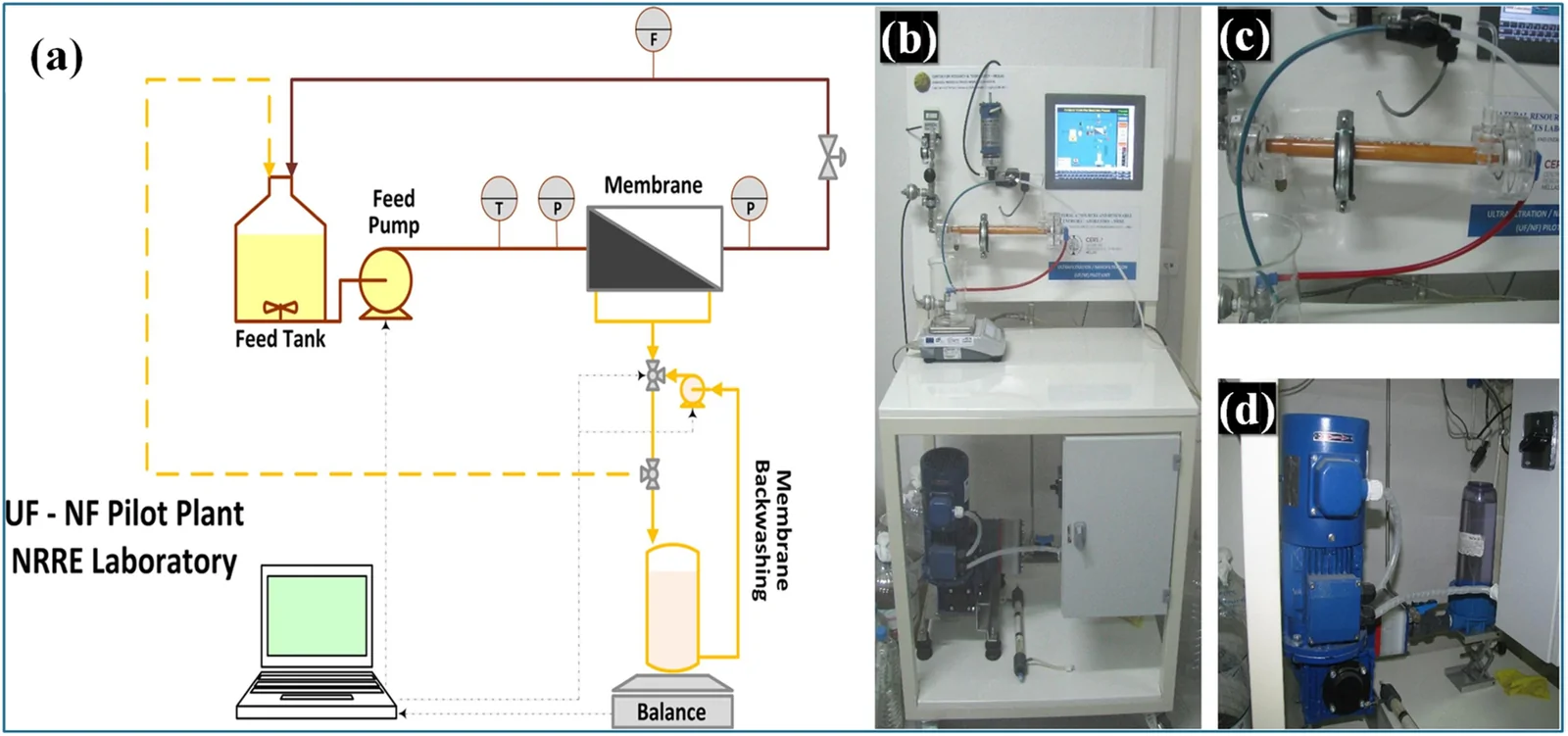
Process flow diagram of the pilot plant for ultrafiltration (UF) and nanofiltration (NF) membrane systems (a); control panel and system setup, showing the operational components including the membrane module (b); close-up view of the membrane module and supporting components (c); feed pump and associated filtration components within the pilot system (d), adapted from ref. 87 with permission from Elsevier K. V. Plakas, A. Mantza, S. D. Sklari, V. T. Zaspalis and A. J. Karabelas, *Chemical Engineering Journal*, 2019, 373, 700–708, copyright 2019.

The field of nanotechnology has also played a pivotal role in the progression of hybrid AOP-membrane systems. Nanomaterials, including carbon nanotubes (CNTs), metal–organic frameworks (MOFs), and nanoscale zero-valent iron (nZVI), have been integrated into membranes, thereby increasing the reactivity and selectivity of AOPs for the degradation of DCF.^88–90^ MIL 88A MOFs were used in membrane systems to enhance the adsorption and degradation of pharmaceuticals like DCF.^91^ MOFs exhibit a substantial surface area and adjustable porosity, enabling them to adsorb a diverse array of contaminants. In hybrid AOP-membrane systems, MOFs serve as catalysts, promoting the decomposition of DCF through oxidative mechanisms while enhancing the selectivity and efficiency of the membrane. The MOF-based membrane demonstrated a rejection efficiency of 80% alongside notable stability and recyclability over 6 operational cycles.

The integration of photocatalysis and membrane separation enabled both contaminant degradation and catalyst recovery within a single continuous process. In addition, these hybrid systems provide several operational advantages, including high water recovery, continuous operation, reduced membrane fouling potential, and minimized chemical consumption, highlighting their potential as sustainable technologies for the treatment of emerging contaminants at larger scales. On the other hand, some limitations of this hybrid treatment include membrane deterioration due to attack by oxidizing groups. This deterioration reduces the membrane's selectivity for the specific contaminant. Furthermore, there is the economic aspect of both the selective membranes and the ozone generators, as well as the LED or UV light sources, which increases both capital and operating costs.

### 4.4. AOPs – biological treatment

Although biological treatment and AOPs present important advantages for wastewater remediation, each technology also exhibits inherent limitations that can restrict its individual performance.^92,93^ Biological treatment methods often show limited efficiency toward persistent pollutants with complex chemical structures, such as DCF. On the contrary, AOPs are temperature-sensitive, costly, and can contribute to secondary pollutants due to incomplete mineralization.

The combination of biological treatment and AOPs has attracted considerable attention as an effective approach for the removal of recalcitrant contaminants from wastewater. Their application seeks to improve contaminant degradation efficiency, decrease treatment time and operational costs, and reduce the generation of secondary pollutants by taking advantage of the complementary strengths of both technologies. These integrated systems are commonly classified according to the arrangement of the biological and oxidation stages. Different configurations have been investigated for the removal of emerging contaminants from wastewater, often achieving enhanced degradation efficiency and higher removal rates to standalone treatment processes. AOPs can be implemented either as a post-treatment or as a pretreatment step, depending on the treatment objectives and effluent characteristics. When applied after biological treatment, AOPs are primarily used to eliminate residual contaminants that remain resistant to biodegradation, thereby improving the final effluent quality.^94^ In conventional hybrid systems, biodegradable organic matter is initially removed through biological treatment; however, pharmaceuticals such as DCF, due to their complex chemical structure and recalcitrant nature, are often only partially degraded during this stage.^95^ To further degrade these persistent compounds and promote the mineralization of remaining organic matter, AOPs such as photocatalysis, ozonation, or photo-Fenton processes are subsequently employed. This sequential approach takes advantage of the complementary strengths of both treatments: biological processes efficiently reduce the bulk organic load, while AOPs selectively target refractory contaminants and oxidation-resistant intermediates, enhancing overall treatment performance and effluent quality. Conversely, when employed prior to biological treatment, AOPs partially oxidize complex organic pollutants into smaller and more biodegradable compounds, facilitating their subsequent biological degradation and enhancing overall treatment efficiency.^95^ AOPs convert DCF into smaller and more easily biodegradable species. This modification, known as a pre-oxidation step, makes the biological treatment more effective by increasing the bacterial degradation of the ligand. This approach can provide a more flexible option to address more toxic and concentrated wastewater.

In both treatment sequences, the hybrid method promotes a more effective removal of DCF from wastewater, resulting in improved treatment performance and greater process sustainability. In a work led by Gimeno *et al.*,^96^ a hybrid method that combines aerobic biodegradation with AOP photocatalysis was employed to treat primary wastewater effluents, composed of a mixture of nine pharmaceutical model compounds, including acetaminophen, caffeine, ketorolac, and DCF, among others (Fig. 6a). The sequential combination of aerobic biological degradation and AOPs enables efficient total removal, even of those pharmaceutical residues that are resistant to biodegradation alone. Application of photocatalytic ozonation also improves degradation rate and mineralization, which extends its usefulness as a reasonable method for the treatment of complex wastewater effluents. Use of sunshine in the hybrid system is both cost-effective and environmentally friendly, especially in tropical regions. This hybrid concept provides a valid solution for the problem of pharmaceutical pollutants in wastewater.

**Fig. 6:**
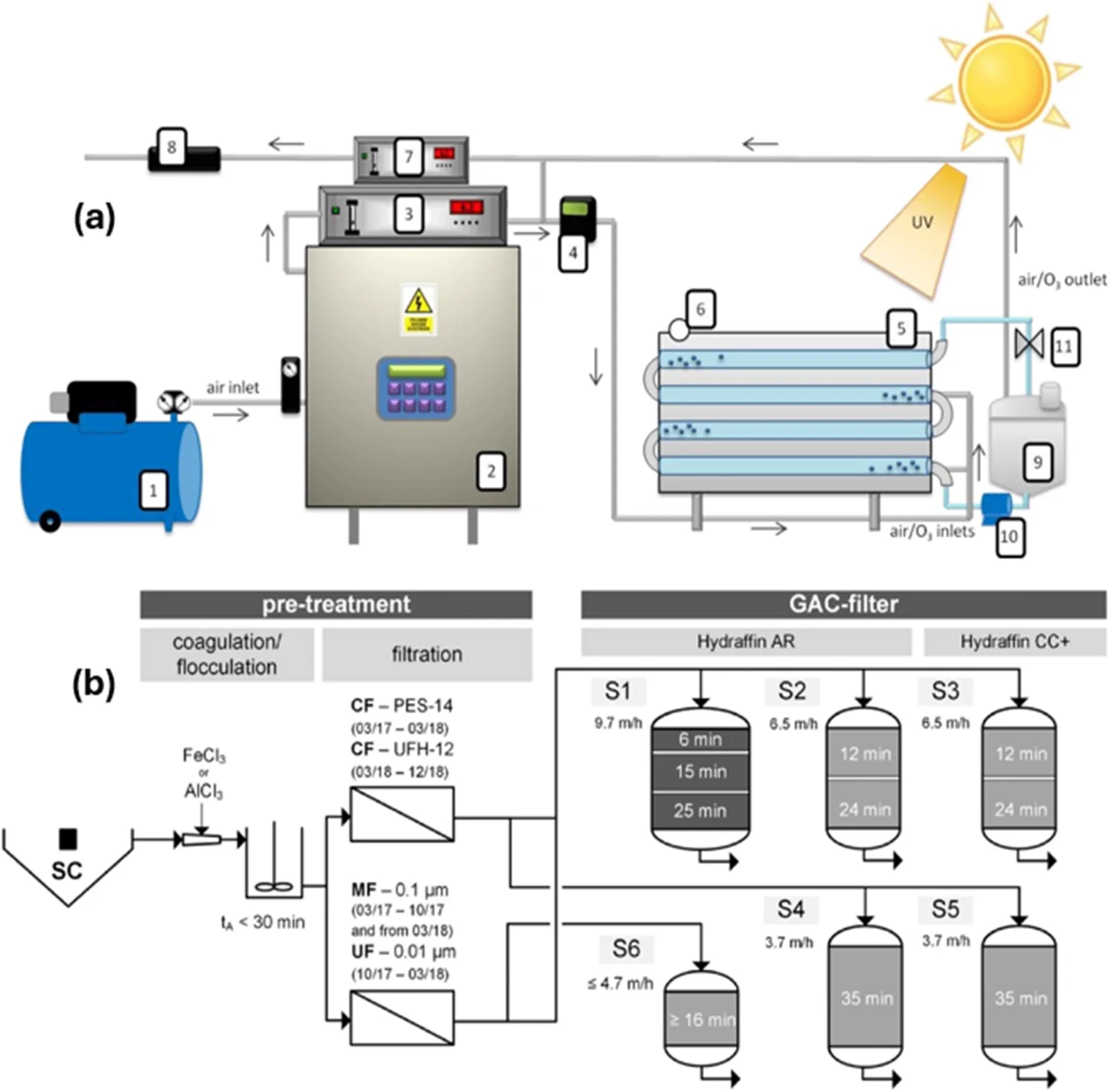
(a) Advanced oxidation treatment setup with solar radiation. (1) Air compressor; (2) ozone generator; (3) ozone analyzer (inlet stream); (4) mass flow controller; (5) CPC reactor; (6) UV radiometer; (7) ozone analyzer (outlet stream); (8) ozone destructor; gas–liquid separator; (10) pump; (11) sample point, reproduced from ref. 96 with permission from Elsevier O. Gimeno, J. F. García-Araya, F. J. Beltrán, F. J. Rivas and A. Espejo, *Chemical Engineering Journal*, 2016, 290, 12–20, copyright 2016. (b) Scheme of the advanced WWTP experimental set up. Pre-treatment (CF: cloth-filtration; MF: microfiltration: UF: ultrafiltration). GAC-filters: full-scale (S1) and pilot-scale (S2–S6) GAC-filters with Hydraffin AR (S1, S2, S4, S6) and Hydraffin CC plus (S3, S5). The EBCTs are cumulative (filters operated in series) and theoretical values. The achieved EBCTs are listed in the SI, reproduced from ref. 98 with permission from Elsevier Fundneider, T., Alonso, V. A., Wick, A., Albrecht, D., & Lackner, S. *Water research*, 189, 116588, copyright 2021.

### 4.5. Biological treatment – adsorption

Integrating biological treatment with adsorption presents a promising approach for achieving enhanced removal of complex pollutants in wastewater. Each treatment technique offers unique benefits: biological processes are effective for breaking down biodegradable substances, while adsorption is well-suited for capturing diverse contaminants on solid surfaces. However, using these methods alone may limit overall effectiveness, particularly with resilient or non-biodegradable compounds. When applied sequentially, biological treatment followed by adsorption can efficiently reduce organic loads and trap residual contaminants that resist biological breakdown. In this configuration, biological treatment initially lowers the concentration of easily degradable pollutants, allowing adsorption to subsequently target and capture the remaining, more resistant compounds. Conversely, the application of adsorption as a preliminary treatment stage can effectively reduce pollutant concentrations and toxicity levels, creating more favorable conditions for microbial activity and improving the subsequent biodegradation of compounds that are otherwise resistant to biological treatment alone. Paredes *et al.*^97^ evaluated the performance of a fluidized-bed aerobic biological activated carbon (BAC) reactor for the removal of several pharmaceuticals, including DCF. The reactor was operated in up-flow mode with hydraulic contact times of 17 and 35 min. At the shorter contact time, removal efficiencies ranged from 70% to 99% depending on the compound, while extending the contact time to 35 min resulted in removal rates exceeding 90% for all pharmaceuticals investigated. These findings highlight the effectiveness of BAC systems for the treatment of pharmaceutical contaminants and demonstrate the positive influence of increased contact time on overall removal performance. Furthermore, T. Fundneider *et al.*^98^ investigated the long-term performance of biologically active granular activated carbon (GAC) filtration for the removal of micropollutants from wastewater over a 32 months operational period (Fig. 6b). Attention was given to the biodegradation of DCF and the influence of biofilm development on filtration efficiency. The results demonstrated that biological activity played a major role in DCF removal, as reflected by the decrease in the normalized concentration (*C*/*C*_0_) from approximately 0.5 to nearly zero during operation. These findings indicate that biodegradation became the dominant removal mechanism once a mature biofilm was established on the GAC surface. The study also highlighted the importance of backwashing for maintaining stable reactor performance and controlling excessive biofilm accumulation. Notably, the required backwashing frequency depended on the pretreatment strategy, with membrane filtration reducing solids loading and consequently requiring fewer backwashing events than cloth filtration.

### 4.6. Other hybrid methods

Recent work cited emerging hybrid approaches for even more efficient DCF decontamination from polluted effluents. The synergistic integration of AOPs with physical techniques such as filtration or separation can be employed to augment the overall effectiveness in this context. Notably, the amalgamation of chemical degradation pathways with physical forces, including ultrasound or magnetic fields, serves to enhance both the reaction kinetics and the overall efficiency of water purification methodologies. Each technique exploits distinct facets of contaminant chemistry, such as solubility, reactivity, and charge, to optimize the degradation of pharmaceuticals like DCF, thereby proposing viable solutions for scalable wastewater treatment.^99–101^

Casierra-Martinez *et al.*^102^ demonstrate the potential of CW-photo-Fenton hybrid systems as an effective strategy for the treatment of pharmaceutical contaminants in wastewater. While the CW-only treatment showed limited performance, with residual DCF concentrations remaining relatively high throughout the operational cycles, all hybrid configurations achieved substantially greater degradation efficiencies and near-complete DCF removal during several treatment stages (Fig. 7a and b). The superior performance of the integrated systems highlights the synergistic effect between biological processes occurring in the wetland and the oxidative degradation promoted by the photo-Fenton reaction. Quang Huy *et al.*^99^ proposed a novel CO_2_-activated hybrid precipitation-adsorption process for the removal of DCF from aqueous media. CO_2_ addition promotes DCF protonation, reducing its solubility and facilitating both precipitation and subsequent adsorption (Fig. 7c and d). Furthermore, CO_2_ activation modifies the charge of the adsorbent surface, enhancing adsorbate–adsorbent interactions and improving overall removal efficiency (Fig. 7e). These results highlight the potential of CO_2_-assisted hybrid systems as an effective approach for the treatment of pharmaceutical contaminants in water. Liu *et al.*,^101^ investigated a hybrid microbubble-enhanced plasma (BE-plasma) system for DCF removal, combining cold atmospheric plasma with microbubble technology to improve the generation and transfer of reactive species. In addition to efficient contaminant degradation, phytotoxicity assays using pea seeds demonstrated that the treated water significantly improved germination and plant growth compared with untreated DCF solutions, indicating a substantial reduction in DCF toxicity (Fig. 7f). These findings highlight the potential of BE-plasma technology as an effective treatment strategy that not only removes DCF but also improves water quality for potential agricultural reuse.

**Fig. 7:**
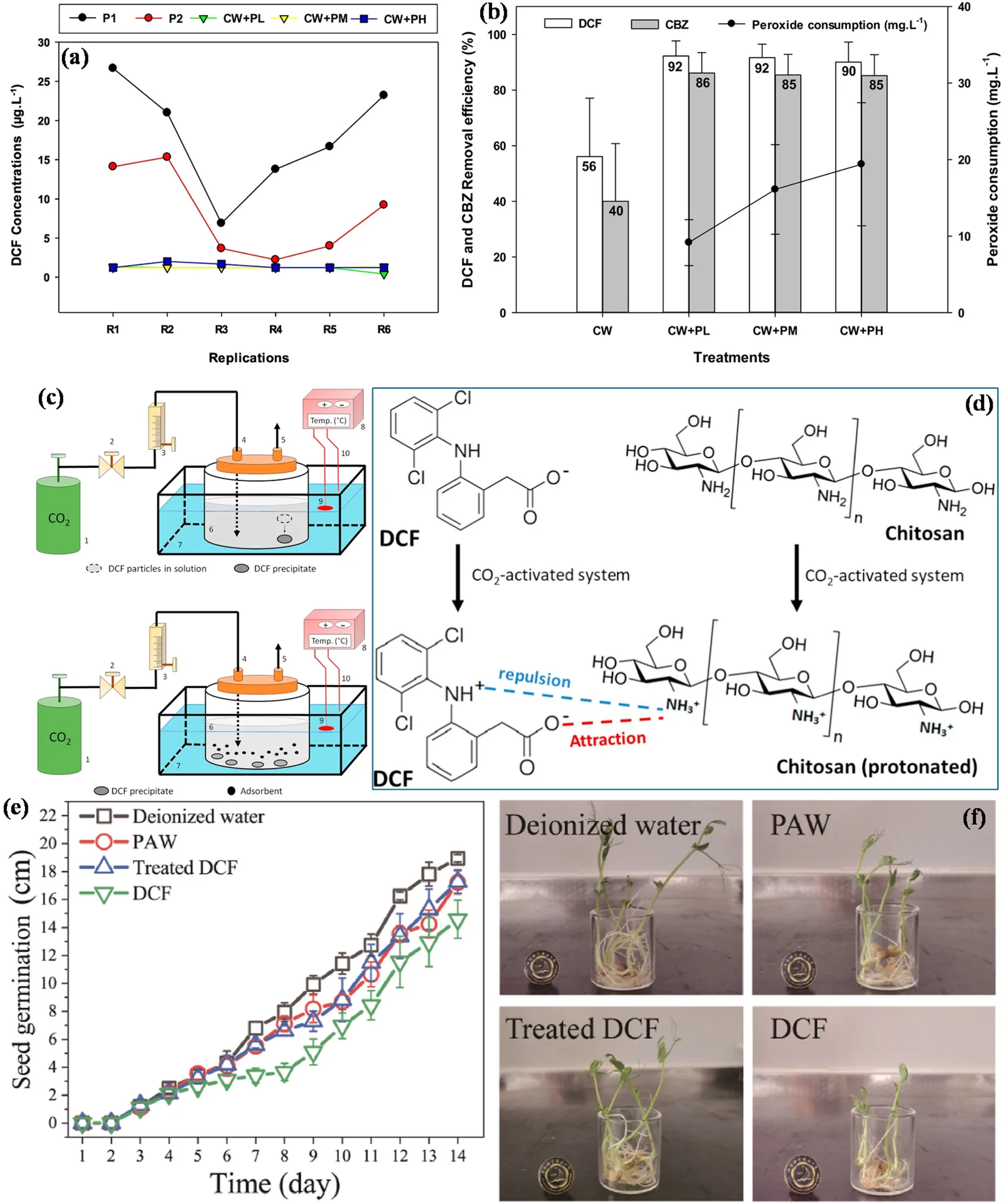
Analysis of DCF and carbamazepine removal efficiencies in Constructed Wetland (CW) coupled systems (a) and (b) behavior of DCF concentrations using solar photo-Fenton treatments with peroxide consumption, adapted from ref. 102 with permission from Elsevier Casierra-Martinez, H. A., Madera-Parra, C. A., Vargas-Ramírez, X. M., Caselles-Osorio, A., & Torres-López, W. A. *Ecological engineering*, 153, 105699, copyright 2020. CO_2_-activated system's two stages: (c) CO_2_-activated precipitation and (d) CO_2_-activated adsorption. The apparatus comprises several essential components: a CO_2_ gas cylinder (1), a CO_2_ pressure regulator (2), and a CO_2_ flow rate controller (3) that ensure precise administration of CO_2_ into the separation vessel (6). A thermostatic water bath (7) sustains the temperature within the system, while the coil heater (10) and temperature control unit (8) furnish supplementary thermal regulation. During the precipitation phase, CO_2_ is infused into the solution, leading to a reduction in the solubility of DCF through the reduction of pH, which consequently prompts the precipitation of DCF, adapted from ref. 99 with permission from Elsevier L. Q. Huy and Y. Shimoyama, *Sep. Purif. Technol.*, 2019, 218, 97–105, copyright 2019. (e) The molecular interactions that are implicated in the adsorption mechanism. (f) The influence of plasma-microbubble treated water on seed germination and plant growth: a comparative examination of DCF-contaminated water, Plasma-treated DCF solution, Plasma-activated water, and deionized water, adapted from ref. 101 with permission from Elsevier Liu, Q., Ouyang, W., Yang, X., He, Y., Wu, Z., & Ostrikov, K. K. *Chemosphere*, 334, 138998, copyright 2023.

Current research reveals significant progress in the design of metal–organic frameworks (MOFs)-based hybrid systems for wastewater treatment.^103–107^ These systems are created by combining MOFs with other functional materials such as graphene oxide,^108–110^ polymers,^111,112^ and MXenes.^113,114^ For instance, Hu *et al.* developed a hybrid membrane composed of polyacrylonitrile (PAN) and MOF-808 for effective DCF removal from water.^115^ The as-developed hybrid membranes provide a high adsorption capacity for DCF, thanks to their remarkable physical characteristics (*e.g.*, large surface area, porous structure, and accessible metal sites). The authors demonstrated that the performance of the MOF-808/PAN membrane stems from the synergistic effect of high surface area and surface charge matching between MOF-808 and DCF. The resulting membrane removes up to 91% of DCF after a single filtration step. Similarly, Gao *et al.* synthesized a hybrid linker-MOF fibrous composite (TCPP@UiO-66/PAN) as a self-cleaning membrane for the efficient removal of DCF.^116^ The TCPP@UiO-66/PAN membrane exhibits a maximum DCF adsorption capacity of 202 mg g^−1^. Furthermore, it demonstrates exceptional dynamic treatment capacity in a series membrane configuration, reaching 3.75 × 10^4^ kg of DCF per kilogram of TCPP@UiO-66/PAN membrane. The combination of adsorption and light-induced AOP (*i.e.*, photosynthesis) enables the efficient regeneration of the saturated TCPP@UiO-66/PAN membrane. This mechanism results in the mineralization of 96% of the adsorbed DCF. Gao *et al.* also fabricated defect-engineered MOF fibers for efficient DCF removal through an adsorption/photodegradation hybrid process.^117^ A high adsorption capacity of 809 mg g-1 is combined with photocatalytic activity within the membrane, enabling the *in situ* degradation of DCF under solar irradiation. This hybrid strategy led to 99% degradation of DCF. In addition, the as-fabricated MOF-based membrane could be reused multiple times while retaining excellent effectiveness. In another study by Ji *et al.*, a bio-based hybrid membrane was developed by incorporating cellulose nanofibers and NH_2_-MIL-101(Fe)-type MOF.^118^ The MOF-based membrane can simultaneously adsorb and degrade DCF in water. The peroxydisulfate activation by this hybrid structure enables synergy between membrane filtration and advanced oxidation. This membrane showed an optimal retention of 54.5% for DCF, coupled with an oxidation performance of 89.5%. Overall, MOF-based hybrid systems represent a promising strategy for achieving superior functionality and performance in treating DCF-laden effluents.

Plasma-based processes represent an innovative approach for removing emerging pharmaceutical contaminants from water.^119,120^ They generate reactive oxidizing species that rapidly break down organic molecules. Lenard *et al.* recently developed an atmospheric-pressure direct-current glow discharge system with a circulating liquid cathode to remove DCF from commercial pharmaceutical formulations (gel and capsule matrices).^121^ This technology achieved a DCF removal efficiency of up to 98%. Phytotoxicity tests after treatment confirmed the absence of phytotoxic effects on seed germination and early growth of Lepidium sativum model plants. Likewise, Wypart-Pawul *et al.* reported that cold atmospheric plasmas are effective for treating DCF and other pharmaceutical contaminants, with removal rates reaching up to 98%.^122^ This performance results from the effects of free radical oxidation, UV radiation, and plasma-induced pyrolysis reactions. Beyond plasma-only systems, researchers have developed hybrid technologies that integrate plasma with advanced oxidation to improve treatment efficiency. Song *et al.* investigated the removal of DCF from simulated wastewater using a plasma-based hybrid system that combined non-thermal plasma, granular activated carbon, and persulfate.^123^ This hybrid process increased DCF removal efficiency (90.4%) compared with plasma alone (66.3%). This enhanced performance was primarily due to the formation of reactive oxygen species (*e.g.*, ˙OH and SO_4_˙^−^), which attack the aromatic structure of DCF. In a study by Liu *et al.*, a hybrid microbubble-cold plasma configuration was developed for DCF removal from wastewater.^124^ The hybrid plasma-bubble system demonstrated a strong synergistic effect, with DCF removal efficiencies up to sevenfold higher than those of the individual systems. In another study, Zupanc *et al.* developed a proof-of-concept by hybridizing sub-atmospheric-pressure plasma and hydrodynamic cavitation for the removal of DCF and other micropollutants.^125^ This study achieved 89 ± 1% DCF removal at 25 °C and 53 W of plasma power. Finally, plasma-based hybrid treatments show great promise for DCF removal from wastewater. However, like any emerging technology, these systems require rigorous pilot-scale testing to assess their real-world feasibility (*e.g.*, long-term operational costs, reactor lifespan, and environmental impacts).

## 5. Critical assessment of hybrid treatment systems

The escalating intricacy of wastewater contaminants necessitates the implementation of hybrid treatment systems that amalgamate diverse removal mechanisms to augment efficiency and address the constraints inherent in single-stage treatments. Table 3 presents a comparative analysis of an array of hybrid technologies that have been investigated for the removal of DCF, elucidating their constituent materials, experimental parameters, and removal efficiency. Herein, we conduct an analysis and comparison of these hybrid methodologies predicated on efficiency, operational practicality, sustainability, and scalability. The selection of a hybrid treatment system is contingent upon several critical factors, such as efficiency, energy consumption, scalability, cost-effectiveness, membrane fouling, maintenance requirements, environmental ramifications, and byproduct generation. Each system possesses distinct advantages and disadvantages that dictate its applicability to specific wastewater treatment scenarios. The efficacy of a hybrid treatment system must be evaluated in conjunction with its energy consumption. This trade-off underscores the significance of selecting technologies that reconcile energy demands with processing duration, particularly within high-throughput wastewater treatment facilities.

**Table 3:** Comparative performance of hybrid treatment systems for DCF removal in wastewater

Hybrid process	Description	Material	DCF concentration/effluent	Experimental conditions	Efficiency	Ref.
Adsorption-AOPs	Combines the adsorption capacity of Fe_3_O_4_@MIL-100(Fe) with H_2_O_2_ activity under visible light	Fe_3_O_4_@MIL-100(Fe), visible light and H_2_O_2_	DCF solution: 60 mg L^−1^	Dark conditions; catalyst dosage: 0.1 g L^−1^; 40 mM of H_2_O_2_	99.4% DCF removal in 3 h; 87.8% TOC removal	126
Adsorption-AOPs	Combines membrane separation, photocatalysis and adsorption operation, all in one unit	Granular activated carbon; TiO_2_ nanoparticles; PVDF flat sheet membranes	DCF solution in Milli-Q water: 4 mg L^−1^	Aeration 0.5 LPM; TiO_2_ 0.67 g L^−1^; pH 6.2; AC coating 23.04 mg cm^−2^	95% DCF and 84% TOC removal in 2 h	127
Adsorption-membrane filtration	Combines PAC adsorption with UF.	PAC and PES membrane	DCF solution in ultrapure water: 0.5 mg L^−1^	PAC dose: 20 mg L^−1^ and 600 L^−1^, respectively. 120 rpm, 20 °C	90% in ultrapure water and 99% in WWTP in 24 h	128
			DCF in the WWTP effluent: 10–600 mg L^−1^			
Adsorption-membrane filtration	Combines PAC adsorption with ultrafiltration by submerged and pressurized method	PAC and PES and PVDF membrane	DCF concentration: 3.5–9.1 µg L^−1^	PAC dosage: 20 mg L^−1^	83% with sPAC/UF in 30 h, and 85% with pPAC/UF in 2 h	75
			Municipal WWTP effluent	Coagulation dosage: 4 mg Fe^3+^·L^−1^		
USAMe	Applies ultrasound, adsorption and membrane filtration simultaneously	PAC and polysulfone membrane	DCF concentration: 10 mg L^−1.^ mixture of pollutants in deionized water	Cross flow configuration. PAC dose: 0.75 g m^−2^. Frequency: 35 KHz	100% degradation in 1 h	129
Adsorption-membrane filtration	Combines PAC adsorption with UF dessinfection process	PAC and PES membrane	DFC concentration: 5 mg L^−1^	Filtration cycle time: 1 h. Operational flux: 87 and 135 L m^−2 .^h^−1^	25–41% in 1 h	74
			Solution in dechlorinated tap water			
Adsorption-membrane filtration	Combines GAC adsorption and membrane filtration	GAC and polyacrylonitrile (PAN) membrane	DCF concentration: 337 ng L^−1^	GAC dose: 10 g L^−1^	99% removal in one day	130
			Reverse osmosis concentrate	Daily replacement of 10% of the GAC		
				10 day continuous experiment		
USAMe	Combines ultrasonic irradiation, adsorption and membrane filtration	PAC, hollow fiber polysulfone membrane	DCF concentration: 0.1–10 mg L^−1^	Cross flow configuration	99% in 30 min	131
			Secondary wastewater effluent	PAC dose: 4.5 g m^−2^		
				Frequencies: 35 and 130 KHz		
Adsorption-membrane filtration	Combines PAC adsorption with UF		DCF 305 ng L^−1^	Flux: 80 L m^−2^ h^−1^. Cycle time: 30 min	88% removal in 30 min and 99.99% removal in 1 h	132
			Municipal WWTP			
Adsorption-membrane filtration	Combines PAC adsorption with UF and coagulants	PAC, PES membrane and FeCl_3_	DCF: 3.08 µg L^−1^	PAC dose: 5 mg L^−1^. Flux 80 L m^−2^ h^−1^	83% in 150 min	133
			Municipal WWTP effluent			
AOP/Photocatalysis + membrane	Hybrid UV/visible photocatalysis combined with filtration using a TiO_2_-modified membrane	TiO_2_-modified membrane	DCF 1.0 µmol L^−1^; aqueous solution	pH 7, UV-LED for photocatalysis, continuous flow	93% degradation after 24 h	84
Fenton-like oxidation + membrane	Fenton-like reaction combined with ceramic microfiltration membrane for degradation	α-Al_2_O_3_/nFe membrane	DCF:1 mg L^−1^; Milli-Q water solution	pH 3, 42.9 mg L^−1^ H_2_O_2_	65% removal and 48% mineralization after 24 h	87
Wetland + solar photo-Fenton	Constructed wetland (CW) coupled with solar photo-Fenton for pharmaceutical removal from domestic wastewater	Fe^2+^/H_2_O_2_	DCF: 15 µg L^−1^; domestic wastewater	Fe^2+^/H_2_O_2_ = 0.1; organic loading rate of 7.2 g DBO_5_ m^−2^ d^−1^; continuous flow rate of 18 L day^−1^; hydraulic retention time of 2.5 days	92% removal in 3 h	102
Plasma-microbubble system	Hybrid cold plasma and microbubble system for DCF degradation in water	Plasma discharge, microbubbles	DCF: 20 mg L^−1^; aqueous solution	6 kV discharge voltage; 2 L min^−1^ gas flow	90.9% degradation after 45 min	101
CO_2_-activated precipitation	CO_2_-activated system for precipitation and adsorption of DCF from water using adsorbents like carbon nanofibers	Carbon nanofibers, chitosan	DCF: 45.5 mg L^−1^; aqueous solution	pH-dependent protonation of adsorbents; 25 °C	93.3% precipitation; 97.6% adsorption efficiency	99
Son-ophotolysis	Combination of ultrasound and UV/H_2_O_2_ in a pilot-scale reactor for synthetic pharmaceutical wastewater degradation	H_2_O_2_, UV light	DCF: 0.25–0.75 mg L^−1^	Ultrasound power 140 W, H_2_O_2_ concentration 1200 mg L^−1^	98% TOC removal after 2 h	134
			Synthetic pharmaceutical wastewater			
Electro-membrane bioreactor	Electrochemical oxidation combined with membrane filtration for DCF and other pharmaceutical removal	GE-Zenon Zee Weed-1 membrane	DCF: 0.01 mg L^−1^; synthetic wastewater	Hydraulic retention time: 19 h; aeration: 98 L min^−1^; current density: 0.5 mA cm^−2^; 5 min ON/20 min OFF	75.25% reduction after 35 days	86
Bio/AOP	Combined biologicaly treated effluent with (V)UV irradiation and ozonation treatment followed by GAC adsorption	(V)UV lamps with *in situ* ozone generation	DCF and seven other targets; biologically treated wastewater	O_3_ dose: 0.030 and 0.045 g O_3_·g^−1^ DOC; residence time: 4.75 min	80–100% degradation in 4.75 min	94
		Granular activated carbon (GAC) columns for post-treatment				
AOP/bio	Ozonation of micropollutants followed by enzymatic post-treatment	Ozone (O_3_) for the initial oxidation of micropollutants followed by laccase enzyme derived from *Trametes versicolor*	DCF: 20 mg L-1; aqueous solution	Ozone capacity: 10 g O_3_·m^−3^; ozonation time: 30 min. Laccase treatment: pH 5 in dH_2_O with 28 µM syringaldazine at 20 °C. Absorbance at 530 nm	100% degradation after 30 min, remain transformation products	95
Adsorption/bio	Granulated activated carbon (GAC) filtration with biological activity	GAC filtration process combines adsorption and biological activity	Wastewater containing micropollutants, dissolved organic carbon	32 months, average inflow: 30–35 m^3^ d^−1^	Nearly complete removal. Better removal was observed at high temperatures	98

### 5.1. Formation and fate of DCF transformation products in hybrid treatment systems

#### 5.1.1 AOPs

The degradation of DCF, particularly during AOPs, inevitably leads to the formation of numerous transformation products (TPs) through reactions such as hydroxylation, dechlorination, decarboxylation, C–N bond cleavage, and aromatic ring opening.^135,136^ Because these pathways depend on the oxidizing species and operating conditions, each AOP generates a distinct TP profile rather than a universal set of intermediates. As an example, Cruz-Muñoz *et al.*,^137^ identified carbazole derivatives during UV photolysis that persist with toxicity levels comparable to the parent DCF. Meanwhile, photocatalysis promotes the formation of phenylacetic acid derivatives which are less persistent and less toxic products. In the case of electro-oxidation, the initial hydroxylation of DCF produces 3- and 4-hydroxy DCF and 4,5-dihydroxy DCF. These hydroxylated derivatives then undergo dehydrogenation to form 5-iminoquinone or decarboxylation to yield (2,6-dichlorophenyl)-indolin-2-one and 2-(2,6-dichlorophenylamine)-benzaldehyde or 4,5-dihydroxy-benzyl alcohol. Finally, the further cleavage of the C–N bond originates monomeric molecules like 2,3-dichloro-4-amino-phenol and 2,6-dichloro-4-hydroxybenzeneamine.^135^

While several TPs are readily biodegradable or further oxidized to low-molecular-weight organic acids, others exhibit greater persistence or toxicity than the parent compound. Ellepola *et al.*,^138^ studied individual DCF photo-degradation products on human kidney (HEK293K) and liver cell lines (HepG2). They reveal that 1-(2,6-dichlorophenyl)indolin-2-one was consistently the most cytotoxic. In HEK293T kidney cells, cell viability decreased by approximately 90%, 80%, and 50% at 250, 125, and 62.5 µM, respectively, indicating markedly higher toxicity than the parent DCF. A similar trend was observed in HepG2 liver cells, where it produced the greatest inhibitory effect, reducing cell viability by approximately 60% and 25% at the highest concentrations tested. Moreover, the 2-(8-chloro-9*H*-carbazol-1-yl)acetic acid, which was the major degradation product, demonstrated dose-dependent toxicity and killed 10% more kidney cells than DCF at the lowest concentration (62.5 µM).

#### 5.1.2 Biological process

In biological processes, microorganisms transform DCF through enzyme-mediated reactions, including hydroxylation, oxidation, dechlorination, decarboxylation, and conjugation, before further degradation of the aromatic ring. These metabolic pathways generate a wide variety of transformation products (TPs), including 4′-hydroxydiclofenac,^139^*N*-(2,6-dichlorophenyl)-2-indolinone,^140^*p*-benzoquinone imine of 5-hydroxydiclofenac,^141^ diclofenac taurine conjugate (DCF-M403), and diclofenac methyl ester (DCF-M310.03),^142^ among many others.^143^ The ecotoxicological profiles of these TPs vary considerably. For instance, 4′-hydroxydiclofenac has been reported to be less toxic than the parent compound.^144^ In contrast, other TPs may retain or even exceed the toxicity of DCF. For example, the methyl ester metabolite DCF-M310.03 exhibited substantially higher toxicity than DCF,^142^ highlighting that biotransformation does not necessarily reduce the environmental risk associated with this pharmaceutical.

#### 5.1.3 Hybrid treatments

Given the large diversity of DCF TPs, toxicity assessment must be performed on a metabolite-by-metabolite basis, which represents a major analytical and ecotoxicological challenge. Consequently, complete mineralization is often considered the most desirable outcome to minimize adverse effects on aquatic ecosystems. In this context, hybrid treatment processes have emerged as promising strategies because they combine complementary mechanisms capable of enhancing DCF degradation beyond that achieved by a single treatment stage.

Hybrid systems generally improve overall removal efficiency, shorten treatment times, and reduce residual toxicity more effectively than standalone biological or physicochemical processes. However, their performance depends strongly on the specific combination of unit operations, since persistent or even newly formed by-products may remain in the treated effluent. The study by Jewell *et al.*^140^ illustrates this complexity by showing that the transformation profile of DCF changes continuously during hybrid treatment. In a hybrid biofilm-activated sludge process, DCF was removed by approximately 88%, while the fate of its TPs varied considerably. Hydroxylated derivatives such as 4′-hydroxydiclofenac and 5-hydroxydiclofenac were substantially reduced (about 83% and nearly complete removal, respectively). In contrast, DCF-lactam, DCF-benzoic acid, TP259 (the result of dechlorination), and TP285 (the result of oxidative ring opening) accumulated during treatment, indicating that the biological process does not simply eliminate DCF but continuously converts it into new intermediates. Therefore, although hybrid biological systems can effectively decrease the concentration of the parent compound, they may simultaneously generate a dynamic mixture of transformation products whose persistence and toxicity must also be assessed. Deeb *et al.*^145^ demonstrated the complementary roles of ozonation and biological post-treatment in removing DCF and its TPs, especially hydroxydiclofenac. Ozonation efficiently degraded the parent compound but simultaneously generated several TPs, which were virtually absent in the influent before ozonation. The subsequent biological treatment further removed these oxidation intermediates, with hydroxydiclofenac showing a substantial decrease or complete disappearance. These results indicate that ozonation acts as a transformation step, converting DCF into more biodegradable intermediates, whereas the biological post-treatment serves as a polishing stage that promotes the degradation of the remaining TPs, thereby reducing their persistence in the treated effluent.

Table 4 summarizes representative hybrid systems evaluated for the treatment of DCF TPs. Overall, these systems exploit complementary mechanisms across successive treatment stages: the first unit promotes the transformation of DCF and its intermediates, while the second stage further adsorbs, biodegrades, or oxidizes the remaining compounds, sometimes leading to near-complete mineralization.

**Table 4:** Transformation products identified during hybrid diclofenac treatment and their subsequent removal

Hybrid system	DCF TPs reported	Second process role	Mineralization achieved?	Reference
Ozonation + constructed wetland	Hydroxy-DCF (mix of 3′,4′,5) at 0.20 µg L^−1^	Retention of residual TP	No, only a reduction of 21%	146
Ozonation + GAC-filter		Retention of residual TP	No, only a reduction up to 90%	
Ozonation + sand/BAC filter		Retention of residual TP	No, only a reduction of 25%	
Ozonation + moving bed biofilm reactor (MBBR)	DCF-amide at 1 µg L^−1^ and 10 µg L^−1^ in laboratory reactors		No, only a reduction of 86% and 59%, respectively	147
	DCF 2,5-quinone imine at 1 µg L^−1^ and 10 µg L^−1^		No, only a reduction of 37% for both concentrations	
	3′-hydroxy DCF at 1 µg L^−1^ and 10 µg L^−1^		Reduction of 86% and 100%, respectively	
Supported liquid membranes + ozonation	5-Hydroxy-DCF at 0.35 mg L^−1^	Degradation of TP	Degradation up to 90%	148

### 5.2. Scalability, real wastewater matrices, and decentralized treatment considerations

Although some hybrid treatment configurations have reported DCF removal efficiencies exceeding 90%, these values should not be directly interpreted as evidence of process scalability. Most laboratory-scale studies are conducted under controlled conditions, including optimized pH, simplified matrices, low hydraulic flow rates, and relatively high DCF concentrations, which may not reflect the operational limitations encountered at larger scales.

In this regard, the assessment of scalability should consider not only changes in operating conditions but also the nature of the matrix being treated.^149^ Unlike synthetic solutions, municipal and hospital wastewater contains a greater diversity and concentration of organic and inorganic matter, as well as other contaminants that can affect the performance of hybrid systems. In the case of adsorbent materials, NOM can compete with DCF for adsorption sites and partially block the porous structure of activated carbon, thereby reducing the availability of sites for DCF uptake. Phal *et al.*^150^ reported that DCF removal on a coal-based activated carbon decreased from 72.7% in ultrapure water to 56.6% in surface water containing NOM, while pre-adsorption of NOM on the activated carbon further reduced DCF sorption. Similarly, membrane fouling can become a major operational limitation in real wastewater, as organic matter and colloidal constituents may accumulate on the membrane surface or within its pores, *via* electrostatic forces and hydrophobic interactions, reducing permeability and increasing cleaning requirements.^151^ In photocatalytic systems, dissolved organic matter (DOM) can also reduce DCF degradation by scavenging the reactive species generated during irradiation. This, in turn, can alter the predominant oxidation pathways and consequently decrease the number of oxidants effectively available for DCF degradation. Gao *et al.*^152^ reported that increasing DOM concentrations progressively inhibited TiO_2_-mediated DCF degradation and decreased the intensity of ˙OH, promoting the formation of O_2_˙^−^ and ^1^O_2_ species. They also demonstrated that the presence of Fe^2+^ and phosphate produced similar inhibitory effects. Therefore, removal efficiencies obtained in synthetic matrices may overestimate the performance of hybrid systems when applied to real wastewater.

For example, Acero *et al.*^128^ studied the performance of a hybrid PAC/UF system for the removal of a mixture of emerging contaminants, including DCF. Their results demonstrated that the system's efficiency strongly depended on the composition of the water matrix. In ultrapure water, a PAC dosage of 20 mg L^−1^ was sufficient to achieve more than 90% DCF removal, whereas a substantially higher dosage (600 mg L^−1^) was required to achieve complete DCF removal in municipal wastewater due to the presence of natural organic matter (NOM) and other competing constituents. The presence of NOM in real municipal effluents can increase the adsorbent demand when adsorption is intended not only for micropollutant removal but also for the removal of competing organic matter and overall improvement of water quality. Furthermore, PAC pretreatment contributed to reducing membrane fouling, demonstrating that adsorption can simultaneously remove micropollutants and condition the water matrix prior to filtration.

An example of the transition from laboratory to pilot scale is the photocatalytic–membrane hybrid reactor developed by Plakas *et al.*,^85^ who implemented a continuous and automated operation unit with a capacity of up to 1.2 m^3^ day^−1^ for DCF degradation through TiO_2_ photocatalysis coupled with ultrafiltration. The study evaluated parameters directly related to scale-up, including hydraulic residence time, TiO_2_ concentration, and UV-C irradiation power. Among these, catalyst loading and UV-C power had a greater influence on DCF removal than hydraulic residence time under the conditions investigated. In addition, the ultrafiltration stage served to retain the catalyst and maintain its presence within the system, although fouling-control strategies involving aeration and periodic backwashing were required. Although these results represent an important step forward compared with laboratory-scale studies, the experiments were conducted using natural feed water, primarily surface water and tap water, whose complexity is lower than that of municipal or hospital wastewater. Therefore, the performance observed at pilot scale cannot be directly extrapolated to more complex wastewater matrices, in which organic matter, ions, and other micropollutants may affect both photocatalytic efficiency and membrane fouling. Another application of hybrid systems at pilot scale was reported by Urtiaga *et al.*,^153^ who evaluated a treatment consisting of UF, RO, and electrochemical oxidation for the removal of pharmaceuticals, including DCF, present in a municipal secondary effluent. While UF achieved less than 20% removal for most micropollutants, the RO stage enabled high retention of the pharmaceutical compounds, concentrating them in a reject stream. The total concentration of micropollutants in this stream was approximately 149 µg L^−1^, and after being subjected to electrochemical oxidation, this concentration decreased to values below 10 µg L^−1^. These results highlight an important advantage of process integration, in which the first stage protects and conditions the membrane feed, while the subsequent stages not only enable contaminant separation but also treat the concentrated stream generated during the process. Moreover, this type of configuration illustrates one of the major challenges associated with the scalability of hybrid systems: high contaminant removal from the main stream does not necessarily imply contaminant destruction, but rather its transfer to a secondary stream that requires additional treatment, thereby increasing the system's energy consumption and operational requirements.

The complex pharmaceutical composition of hospital wastewater and pharmaceutical industrial effluents may limit the capacity of biological systems to degrade recalcitrant compounds by altering microbial growth, community structure, and metabolic activity. For example, the presence of xenobiotic compounds can exert toxic or inhibitory effects on microorganisms, leading to lower microbial diversity and richness and alterations in nitrifying communities in industrial wastewater treatment plants.^154,155^ In real wastewater matrices, this inhibition may be further intensified by the coexistence of natural organic matter (NOM), inorganic ions, surfactants, and other pharmaceuticals, which can modify substrate availability, microbial interactions, and the physicochemical conditions of the biological system.

Consequently, the performance observed with synthetic or simplified matrices may overestimate the capacity of biological processes to transform recalcitrant pharmaceuticals under real operating conditions. A hybrid configuration can partially mitigate these limitations of a single microbial community by providing complementary microbial niches and distributing the treatment load among microbial communities with different physiological and metabolic capabilities. Escola Casas *et al.*^156^ evaluated a pilot-scale hybrid system consisting of activated sludge, Hybas reactors, and a polishing MBBR, operated for 10 months with hospital wastewater. The authors observed that organic matter removal and nitrification occurred mainly in the first activated sludge reactor, whereas attached biomass was more effective at removing pharmaceuticals. The coexistence of suspended and attached biomass across different treatment stages favored the establishment of microbial communities with distinct metabolic capabilities, which may increase the resilience of the system to the complex composition of hospital wastewater. The configuration of the hybrid process does not eliminate the inhibitory effects of complex wastewater matrices, it provides complementary metabolic capabilities, allowing the system to maintain contaminant removal even when some microorganisms are affected by toxic pharmaceuticals or other matrix components. High concentrations of inhibitory compounds may still reduce microbial activity, disrupt community stability and nitrification, and consequently decrease pharmaceutical biodegradation.^157^ Thus, the main advantage of hybrid systems is their greater resilience to the variability and inhibitory effects of real wastewater, rather than complete resistance to these conditions.

Table 5 shows some metrics used to scale up hybrid treatment systems for DCF removal at laboratory, pilot, and full-scale levels. These studies illustrate that scale-up involves more than maintaining high contaminant removal. In real-water applications, hybrid systems must simultaneously address matrix complexity, membrane protection, hydraulic performance, energy requirements, and the management of secondary concentrated streams.

**Table 5:** Scalability metrics of hybrid treatment systems for DCF removal at laboratory, pilot, and full-scale levels^a^

Hybrid technology	Scale	Wastewater type and DCF concentration	DCF removal	Operational conditions	Energy/economic metric	Ref.
TiO_2_ nanotubes/MF photocatalytic membrane + UV	Laboratory	Synthetic DCF solution at 5 mg L^−1^	DCF removal: 94% after 240 min in the static experiment; and complete degradation was demonstrated in the continuous setup	Operation time: 10 days; membrane flux: 88 mL min^−1^·cm^−2^ bar^−1^; operating pressure: 800 mbar; radiant flux density 7.6 mW cm^−2^	NR	158
UF + RO + electro-oxidation	Pilot	Municipal WWTP secondary effluent; real wastewater; DCF among 12 target pharmaceuticals	UF < 20% for most micropollutants; high pharmaceutical rejection by RO; >93% reduction of total micropollutants in RO concentrate after electrooxidation	WWTP capacity: 110 000 m^3^ day^−1^ at 85% capacity; UF unit flow rate: 2.5 m^3^ h^−1^ average; RO feed flow rate: 2.4 m^3^ h^−1^ constant; electrooxidation volume: 2 L working volume with 10 L min^−1^ recirculation	NR	153
Adsorption + photocatalysis + membrane separation + oscillatory flow in a single APC reactor	Laboratory	Synthetic DCF solution at 4 mg L^−1^ at the reactor inlet	DCF removal: 95%; TOC removal: 84% in 2 hours	Operation time: 2–100 h; treatment capacity: 21.6 L day^−1^; HRT: 39 min; TiO_2_ concentration: 0.67 g L	Energy consumption: 55–109 kW h m^−3^·order; treatment cost: 2.2–4.4 $·m^−3^	127
PAC/UF + H_2_O_2_-based AOP + UF	Pilot	Controlled water matrix; DCF concentration: 1.0 mg L^−1^	DCF removal: complete degradation; TOC removal: up to ∼47% after 4 h during the AOP regeneration experiments	Total effective volume: 25L; treatment capacity: 1.2 m^3^ day; HRT: 33 min; H_2_O_2_ concentration: 10–80 mg L^−1^. Regeneration time: 180 min	NR	159

a NR: Not reported; HRT: hydraulic retention time.

Although centralized hybrid treatment systems can handle large flow rates and control a wide range of pharmaceutical contaminants present in municipal effluents, their application involves treating DCF once it is highly diluted and mixed with other compounds in the matrix, which may increase the treatment demand and hinder its selective removal. In this context, point-source treatment may be particularly attractive for hospital wastewater or pharmaceutical industry effluents because of the higher concentration and diversity of pharmaceuticals present compared with municipal streams.^160^

Prasertkulsak *et al.*^161^ demonstrated the feasibility of decentralized treatment at pilot scale by operating a 1.3 m^3^ MBR directly with hospital wastewater using a hydraulic retention time of 3 h. The system enabled the removal of several pharmaceuticals and showed that, even under a short HRT, the removal of some compounds, including DCF, was associated with both biodegradation and adsorption onto colloidal particles subsequently retained by the membrane. These results support the potential of MBRs as a point-source treatment barrier capable of reducing the load of DCF and other pharmaceuticals before their discharge into the municipal sewer system. However, the dependence of treatment performance on the characteristics of each contaminant indicates that MBRs do not guarantee complete micropollutant removal, and therefore the integration of subsequent advanced treatment stages is necessary to achieve further removal. Pariente *et al.*^162^ reviewed different strategies for the on-site treatment of hospital wastewater and showed that integration with AOPs and MBRs can enhance the removal of recalcitrant compounds, including DCF, while reducing oxidant demand compared with the direct application of these processes. However, although AOPs can achieve high removal efficiencies at laboratory scale, their implementation at pilot and full scale remains limited and challenging because of reagent and energy requirements, as well as the need for operational control.

These studies indicate that point-source treatment can be considered a complementary strategy to centralized treatment systems by reducing the contaminant load before its introduction into the sewer network and potentially improving the performance of advanced treatment stages at centralized facilities. Nevertheless, its sustainability depends on the availability of infrastructure, technical capacity, and resources to operate advanced systems in a decentralized manner.^163^ In addition, variability in the number of hospitals, effluent flow rates, and discharge characteristics further complicates its implementation as a standalone strategy.

Finally, investigations should also explore integrating real-time monitoring and process control into hybrid treatment systems to improve operational stability under variable wastewater conditions. The incorporation of online sensors and adaptive control strategies may enhance process robustness and resource efficiency.^156^ Future sensor-enabled hybrid systems should integrate influent and effluent water-quality indicators with process-specific measurements. Validated rapid DCF assays or site-calibrated UV254/fluorescence surrogates could be combined with DOC, turbidity, conductivity, pH, oxidation-reduction potential, oxidant residual, UV dose, membrane flux/transmembrane pressure, dissolved oxygen, ammonia, and respirometric activity.^157,158^ These measurements could support feedback control of chemical dose, irradiation power, hydraulic residence time, backwashing, and biological loading. However, direct evidence that DCF-specific adaptive control reduces lifecycle cost relative to a well-optimized fixed regime remains limited; surrogate models require matrix-specific calibration and periodic confirmation by LC-MS/MS.

### 5.3. Regulatory compliance and management of secondary waste streams

Regulatory benchmarks for DCF remain heterogeneous. In the European Union, Directive (EU) 2026/805 establishes annual-average environmental quality standards for DCF of 0.040 micrograms per liter (40 ng L^−1^) in inland surface waters and 0.004 micrograms per liter (4 ng L^−1^) in other surface waters. These values are receiving-water quality standards, not uniform end-of-pipe effluent limits.^164^ Separately, Directive (EU) 2024/3019 on urban wastewater treatment requires quaternary treatment at designated plants and evaluates at least 80% average removal across a specified group of indicator micropollutants that includes DCF; it does not impose an 80% DCF-specific removal requirement or a universal DCF effluent concentration.^165^ In the United States, DCF is generally addressed through contaminant-of-emerging-concern monitoring and permit-specific studies rather than a nationwide numeric effluent limit.^166^ Consequently, percentage removal alone cannot demonstrate compliance with an ultra-low receiving-water target. The removal required to reach a target concentration depends on the influent concentration [*R*_required_ = 1 − (*C*_target_/*C*_0_)]; for example, an influent concentration of 1 microgram per liter would require 96% removal to reach 40 ng L^−1^, whereas 10 micrograms per liter would require 99.6%. Most laboratory studies reviewed here do not demonstrate reliable attainment of these endpoints in continuous real-wastewater operation because they report percentage removal from relatively high initial concentrations, often without sufficiently low analytical quantification limits. Regulatory assessment should therefore report influent and effluent concentrations, method quantification limits, temporal variability, transformation products, and toxicity rather than removal percentage alone.^167^ Moreover, membrane retentate, spent adsorbents, regeneration eluates, and treatment sludge may contain the contaminant mass removed from the aqueous phase; therefore, separation must not be equated with destruction.^168^ Scale-up should include a complete mass balance and a residual-management plan addressing waste classification, leaching and ecotoxicity, regeneration streams, catalyst or metal release, concentrate treatment and final disposal or destruction in accordance with the applicable waste-management framework.^169^

## 6. Life cycle impact and commercially oriented benchmarks

Life Cycle Assessment (LCA) serves as an essential instrument for scrutinizing the environmental sustainability of wastewater treatment methodologies. Through the evaluation of raw material extraction, energy utilization, emissions, and waste generation, LCA furnishes a holistic framework for the comparative analysis of various treatment processes aimed at the removal of DCF. Within hybrid wastewater treatment systems, which amalgamate adsorption, AOPs, membrane filtration, and biological treatments, an LCA facilitates the quantification of trade-offs between operational efficiency and ecological impact.^170,171^

This assessment compares key sustainability metrics, including carbon footprint, energy consumption and waste production, between different wastewater treatment methods. Traditional biological treatments, such as activated sludge systems, consume less energy but struggle to completely remove DCF.^172^ Hybrid systems, on the other hand, which incorporate adsorption, AOPs, and membrane filtration, achieve higher removal efficiencies but require more energy and chemical inputs.^173^ Published LCAs have been used to examine whether the environmental benefits of improved contaminant removal compensate for the additional energy, material, and chemical requirements. In addition, this LCA identifies the environmental burdens at each phase of processing, including chemical use, materials production, operational efficiency and waste generation. Many hybrid methods use chemical oxidants such as hydrogen peroxide, ozone and persulfates, whose production and transport have indirect environmental impacts. To ensure a systematic analysis, the system boundaries for this LCA encompass three principal life cycle stages: raw material extraction and production, operational phase, and end-of-life management (Fig. 8a). The raw material extraction phase entails the production and transportation of treatment materials such as adsorbents (*e.g.*, activated carbon, biochar, MOFs), catalysts (*e.g.*, iron, titanium dioxide), and membranes. This stage is pivotal in determining the embodied carbon and energy footprint associated with the materials employed in hybrid wastewater treatment.^174^ The operational phase addresses the electricity consumption requisite for AOPs, membrane filtration, UV-LEDs, and electrochemical processes, in addition to the utilization of chemical reagents, including oxidants and cleaning agents. Emissions stemming from energy-intensive processes and the potential liberation of intermediate degradation products are also considered. Lastly, the end-of-life management phase concentrates on the disposal and prospective regeneration of spent adsorbents, the handling of membrane waste, and the treatment of sludge and other secondary pollutants.^175^

**Fig. 8:**
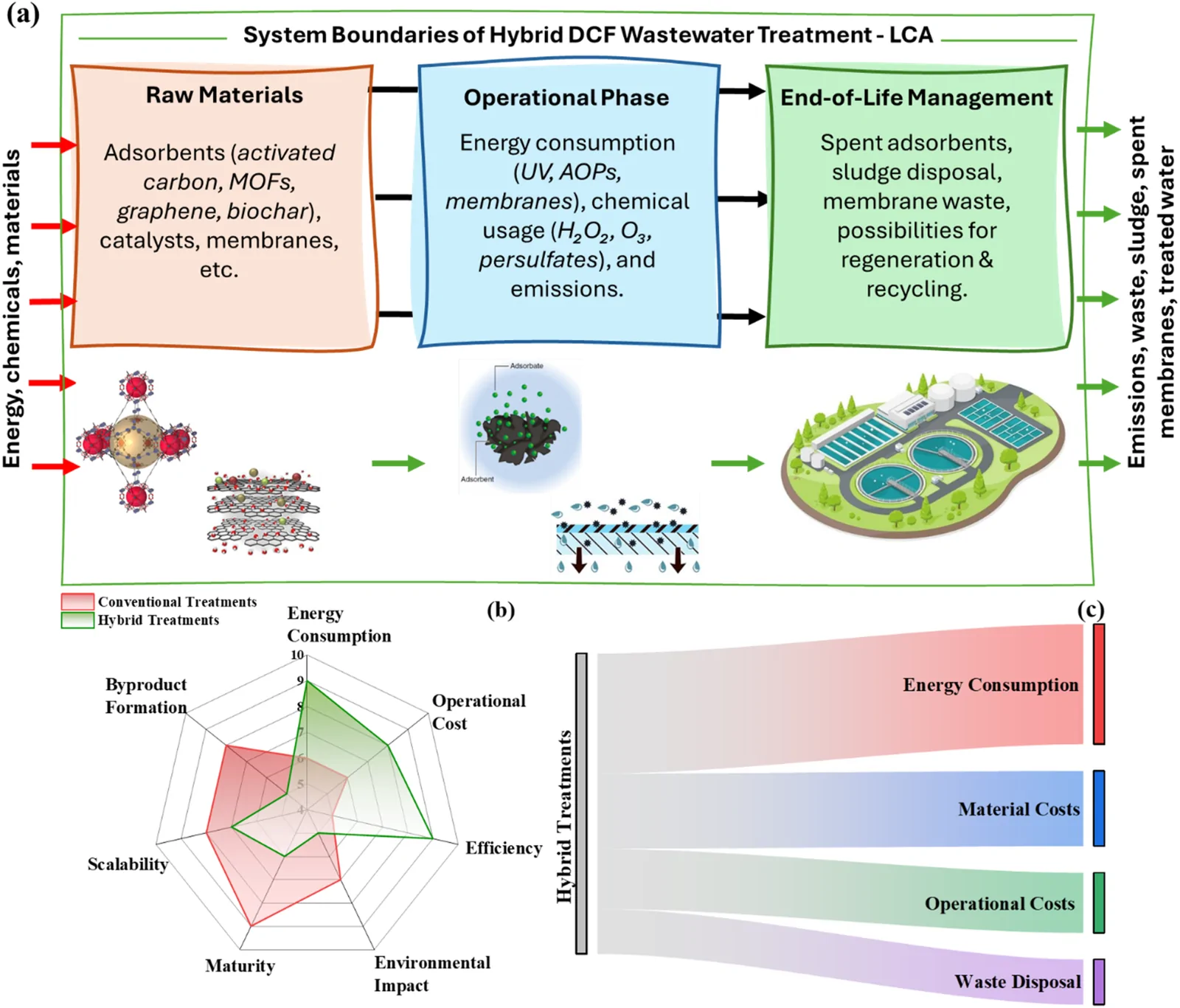
System boundaries of hybrid DCF wastewater treatment (a). Performance comparison of conventional *vs.* hybrid wastewater treatment (b). Resource allocation in hybrid wastewater treatment processes (c).

A direct comparison of the life-cycle performance of adsorption-regeneration and destructive oxidation pathways requires a common functional unit, equivalent treatment targets, consistent system boundaries, and the same life-cycle impact assessment method. These conditions are rarely satisfied across published DCF studies. Consequently, values obtained from different LCAs should not be pooled or used to produce an absolute ranking without accounting for differences in DCF concentration, water matrix, removal target, treatment scale, electricity mix, and inclusion or exclusion of transformation products. Nevertheless, available studies reveal consistent contrasts between adsorption-based and destructive treatment pathways.

Zepon Tarpani and Azapagic^176^ compared GAC adsorption with regeneration, solar photo-Fenton, and ozonation using the treatment of 1000 m^3^ of secondary effluent as the functional unit. DCF, present at a mean concentration of 0.65 µg L^−1^, was included among nine pharmaceutical and personal care products. The GAC scenario included thermal regeneration, a 10% carbon loss during regeneration, and final disposal after ten regeneration cycles. Under the mean operating conditions, the global warming potentials of GAC, solar photo-Fenton, and ozonation were 252, 249, and 316 kg CO_2_-eq per 1000 m^3^, respectively. Their corresponding fossil-resource depletion potentials were 88, 81.5, and 97 kg oil-eq per 1000 m^3^. Fossil-resource depletion is reported here as an energy-resource indicator, but it should not be interpreted as being numerically equivalent to cumulative energy demand. Although GAC and solar photo-Fenton exhibited similar climate-change results, their environmental hotspots differed substantially. For GAC, virgin activated-carbon production accounted for 41% of the climate-change impact and regeneration energy for another 26%. Fresh activated-carbon production and regeneration also contributed 50% and 24%, respectively, to fossil-resource depletion. The principal burdens of the adsorption-regeneration pathway therefore arise from carbon precursor production, activation, regeneration energy, adsorbent losses, transportation, and final management of spent carbon. For solar photo-Fenton, approximately 50% of the climate-change impact originated from H_2_O_2_ production and 47% from other operational inputs. Ozonation exhibited the highest climate-change and fossil-resource burdens, with electricity accounting for 71% of its climate-change impact and 75% of its fossil-resource depletion. These results show that adsorption primarily transfers DCF and other micropollutants to a solid phase, whereas AOPs can destroy the parent compounds but shift a considerable burden toward electricity and reagent production.

Freshwater ecotoxicity outcomes further demonstrate that a high removal percentage does not automatically translate into a net life-cycle benefit. In the same comparative LCA, GAC required a pharmaceutical removal efficiency greater than 65%, and ozonation at least 75%, for their life-cycle freshwater ecotoxicity to become comparable to that of secondary effluent without advanced treatment. Solar photo-Fenton achieved a lower freshwater ecotoxicity only under the minimum operating-input scenario and at removal efficiencies above 85%. These results apply to the modeled mixture of nine pharmaceuticals and should not be interpreted as DCF-specific thresholds. They also remain sensitive to the toxicity characterization factors and the number of parent compounds and transformation products included in the inventory.

A DCF-specific ex-ante LCA was conducted by Pizzichetti *et al.*^171^ using the treatment of 1 L of water containing 20 mg L^−1^ DCF to achieve 90% removal as the functional unit. In the UV-C LED photoreactor, the climate-change impact was approximately 0.0352 kg CO_2_-eq per functional unit, with more than 97% associated with electricity consumption. The addition of H_2_O_2_ reduced the average impacts across the evaluated categories by approximately 35% compared with UV-C photolysis because the shorter irradiation time outweighed the upstream impact of H_2_O_2_ production. The contribution of H_2_O_2_ production remained below 1% under the tested conditions. UV-C/free-chlorine treatment also shortened the treatment time but exhibited higher impacts than UV-C/H_2_O_2_, particularly for ozone depletion and freshwater ecotoxicity. Replacing the conventional electricity mix with photovoltaic and wind electricity reduced the climate-change impact by approximately 80% and 93%, respectively.

The absolute values of this DCF-specific study cannot be compared directly with those of the municipal-effluent study because the former used a laboratory-scale synthetic solution containing 20 mg L^−1^ DCF and a cradle-to-gate boundary, whereas the latter evaluated a mixture of pharmaceuticals at environmentally relevant concentrations and included infrastructure, operation, regeneration, waste management, and decommissioning. The DCF-specific LCA also evaluated removal of the parent compound rather than complete mineralization and did not provide a cradle-to-grave toxicity assessment of all transformation products. It is therefore more appropriate for hotspot identification than for establishing the full-scale superiority of UV-based AOPs.

The identified hotspots provide specific directions for sustainable process engineering. For adsorption-based systems, the priority measures are the use of locally sourced and low-impact carbon precursors, reduction of adsorbent dosage, extension of bed life, minimization of carbon loss during regeneration, use of renewable energy or waste heat for regeneration, and destructive treatment of the concentrated regeneration stream. For AOPs, prioritize low-carbon electricity, energy-efficient UV-LEDs and ozone generators, improved photon and ozone-transfer efficiency, real-time oxidant dosing based on water quality, and biological pretreatment to reduce dissolved organic carbon and oxidant demand. Combining adsorption with targeted oxidation of the smaller regeneration stream may reduce the energy required to oxidize the entire wastewater volume while preventing DCF from being transferred indefinitely between environmental compartments. However, this benefit must be confirmed through a harmonized cradle-to-grave LCA that includes adsorbent production and regeneration, catalyst or membrane replacement, residual DCF, transformation products, toxicity, sludge, and final disposal.

### 6.1. Commercial feasibility and industrial scalability

The global market for DCF has been witnessing consistent growth. In 2024, the market was estimated to be valued at approximately $3.99 billion, with forecasts suggesting a compound annual growth rate (CAGR) of 4.40%, potentially culminating at $6.14 billion by 2034.^177^ The increasing prevalence of chronic pain conditions like arthritis and musculoskeletal disorders, particularly among the aging population, has led to widespread DCF use. As a result, DCF is frequently detected in wastewater, raising environmental concerns due to its persistence and ecological risks. To address this issue, advanced wastewater treatment technologies are needed to remove DCF efficiently. Hybrid treatment strategies, combining adsorption, membrane filtration, AOPs, and biological treatments, have shown high removal efficiencies, with some methods achieving up to 100% elimination under optimized conditions.^27^ The commercial viability and scalability of hybrid wastewater treatment technologies depend on several key factors. These methods require high capital and operating investments, as alternative processes have a high energy demand, which increases operating costs, while membrane technologies require regular maintenance and replacement. Integrating these advanced processes into existing treatment plants can be difficult and costly, often requiring substantial modifications. However, stringent environmental regulations are encouraging the adoption of hybrid treatments to ensure the elimination of new contaminants such as DCF. In addition, growing public awareness and the demand for cleaner water resources are driving industries to implement more efficient wastewater treatment solutions.^178,179^

Fig. 8b compares conventional and hybrid wastewater treatment methods based on key performance criteria, highlighting their trade-offs. Hybrid treatments are more effective at removing DCF as they combine complementary mechanisms. However, these systems consume significantly more energy due to the integration of AOPs or the operational demands of membrane filtration, particularly at large scale. They also have higher operating costs due to maintenance and chemical use. However, hybrid systems reduce environmental impact by improving pollutant degradation and minimizing harmful by-products. Conventional treatments consume less energy and are more cost-effective, but present higher environmental risks due to incomplete disposal of pharmaceuticals. Scalability favors conventional methods, as they are widely implemented in existing infrastructures, whereas hybrid systems require further adaptation and investment. Conventional technologies remain more mature, having been extensively optimized, while hybrid methods are still under development and require further research for widespread adoption.

### 6.2. Market-ready hybrid treatments

The transition from laboratory-scale inquiry to commercially viable hybrid wastewater treatment technologies mandates a balance between high removal efficiency, cost-effectiveness, and industrial scalability. Several hybrid methodologies have emerged as market-ready solutions for the extraction of DCF and other pharmaceutical contaminants from wastewater, amalgamating adsorption, membrane filtration, AOPs, and biological treatments. These technologies provide enhanced contaminant removal while striving to alleviate operational costs and reduce environmental impact. The commercialization of hybrid DCF removal technologies necessitates a structured transition from laboratory-scale research to full-scale industrial deployment. This process encompasses several critical stages, including technology optimization, pilot-scale demonstration, regulatory approval, economic feasibility assessment, and integration into existing wastewater treatment infrastructure.

Hybrid wastewater treatment strategies typically initiate with laboratory-scale investigations, wherein variables such as adsorbent materials, oxidant dosages, catalyst efficacy, and membrane configurations are fine-tuned to achieve optimal DCF removal.^27^ However, the transition from controlled laboratory environments to real-world wastewater matrices presents complexities, including the presence of co-contaminants, variations in water chemistry, and challenges pertaining to operational stability.^25^ To address this disparity, pilot-scale trials must be executed within municipal or industrial wastewater treatment facilities, enabling researchers to validate efficacy under continuous flow scenarios. Recent investigations have demonstrated that pilot-scale AOP-membrane hybrid systems can attain 95–99% DCF removal; however, energy consumption continues to pose a significant challenge.^85^

Fig. 8c illustrates the allocation of resources and distribution of costs in hybrid wastewater treatment systems. Energy consumption is the biggest expense, mainly due to alternative processes and membrane filtration, which pose problems for large-scale implementation. Materials costs (*e.g.* adsorbents, catalysts, membranes) also contribute significantly to overall expenditure. Operating costs, including maintenance, monitoring and chemical replenishment, add to the financial burden. Although waste disposal costs are relatively low, they remain important for long-term sustainability. To improve commercial viability, strategies such as improving energy efficiency, increasing membrane durability and regenerating adsorbents are essential. In addition, compliance with environmental regulations is essential for successful industrial adoption. Stricter pharmaceutical discharge limits, as established by the EU Water Framework Directive and the US EPA, are driving the demand for advanced wastewater treatment technologies.^26^ However, many hybrid systems lack standardized approval protocols due to their multi-step treatment processes. For industrial-scale adoption, hybrid solutions must be compatible with existing wastewater treatment infrastructure. Conventional activated sludge systems, widely used in municipal and industrial facilities, often struggle with refractory contaminants like DCF due to low biodegradability.^11^ Hybrid solutions, such as AOP-biological or adsorption-membrane systems, can serve as tertiary treatments, improving removal efficiency without requiring a complete overhaul of current facilities.^67^

### 6.3. Economic and environmental benchmarking

Beyond mere removal efficiency, the effectiveness of hybrid wastewater treatment systems in addressing pharmaceutical contaminants such as DCF is contingent upon various factors, including energy consumption, cost per cubic meter treated, operational duration, and scalability. This section delivers a comparative evaluation of selected hybrid technologies, utilizing empirical data derived from published literature and market analyses, encompassing critical performance metrics and life-cycle sustainability indicators. The amalgamation of economic and environmental metrics, such as LCA impact, investment expenditures, and payback periods, facilitates a more nuanced appraisal of a system's feasibility for deployment. For example, PAC/UF systems exhibit moderate efficiency (∼85% DCF removal) but are characterized by low energy consumption (0.056 kWh m^−3^) and brief operational periods, rendering them appropriate for municipal contexts with cost constraints.^75^ Conversely, high-performance advanced oxidation process (AOP)-membrane systems achieve removal efficiencies exceeding 95% yet may require energy inputs greater than 0.2 kWh m^−3^ (ref. 85) thereby raising operational expenditure (OPEX) concerns. It is noteworthy that CO_2_-activated hybrid systems present a cost-effective, low-energy alternative with elevated removal efficacies (>97%) and simplified infrastructural requirements^99^ thus making them appealing for decentralized implementations. Technologies such as electro-membrane bioreactors and granular activated carbon/bio-activated carbon (GAC/BAC) systems exemplify a judicious equilibrium between operational efficiency and ecological ramifications, benefiting from low LCA scores and extended service lifespans. Table 6 offers a multi-criteria comparison of ten hybrid systems across dimensions of efficiency, energy consumption, cost, scalability, and LCA. These criteria enable the identification of technologies that are optimally aligned with environmental, economic, and regulatory frameworks.

**Table 6:** Multi-criteria benchmarking of hybrid wastewater treatment systems for DCF^a^

System	Matrix and evidence scale	Reported DCF performance	Demonstrated capacity (m^3^ d^−1^)	Energy (kWh m^−3^)	Footprint evidence	TRL^b^	Refs
PAC/UF (pressurized)	Real municipal WWTP effluent; continuous pilot operation for 6 months	80–95% for both configurations; pressurized PAC/UF was slightly higher	NR; flux to 80 LMH (pPAC) and 23 LMH (sPAC)	0.056 pPAC; 0.178 sPAC (study estimates)	NR	**6**	75
GAC + UV/TiO_2_ membrane	Real river and tap water; continuous, automated pilot PMR	Complete disappearance at 0.05 mg L^−1^ in surface water; 82.3–85.8% in selected tap-water tests; TOC removal 20–40%	1.2 maximum; 25 L reactor and 4.19 m^2^ UF	NR; 3.12 calc., lamp electricity only at maximum flow	NR	**6**	85
Fe_3_O_4_@MIL-100 + AOP	Controlled aqueous DCF solution; laboratory batch	99.4% DCF removal and 87.8% TOC removal after 12 h	NR	NR	NR	**4**	126
CO_2_-activated adsorption	Controlled aqueous DCF solutions; laboratory batch	93.3% maximum precipitation; 97.6% CNF polishing; final DCF <0.115 mg L^−1^. The 97.6% value is not overall train removal	NR	NR	NR	**4**	99
Electro-membrane bioreactor	Synthetic municipal wastewater; continuous laboratory reactor	75.25 ± 8.79%; conventional MBR control 50.09 ± 11.02%	0.0164 calc. From 13 L reactor and 19 h HRT	NR	NR	**4**	86
PAC-UF with FeCl_3_	Spiked, dechlorinated tap water; one-hour laboratory cycles	25–41% at 5 mg L^−1^ PAC; up to 57% at 15 mg L^−1^ PAC	NR; 0.32 or 0.50 L min^−1^ during 60 min cycles	NR	NR	**4**	74
GAC-BAC bioreactor	Real WWTP effluent; one full-scale and five pilot filters; monitored to 32 months	Usually >90% during the stable period, with compound-specific breakthrough and variability	30–35 treatment facility	NR	NR	**7**	98
Plasma-Microbubble	Synthetic DCF-contaminated water; laboratory batch	90.9% after 45 min under optimized conditions; salts and humic acid were also tested	NR	NR	NR	**4**	101
Constructed Wetland-Photo-Fenton	Real domestic wastewater spiked to 15 µg L^−1^; outdoor hybrid experiment at a WWTP	Up to 92% overall; wetland stage 56%; photo-Fenton stage 78–79%	CW 0.018; photo-Fenton 0.003 per 3 h batch; train not synchronized	NR	Full train NR; CW 0.375 m^2^, equal to 20.8 m^2^ (m^3^ d^−1^) calc	**5**	102

a NR, not reported in the cited paper; NC, not calculable from the reported information; PAC, powdered activated carbon; UF, ultrafiltration; PMR, photocatalytic membrane reactor; CNF, carbon nanofiber; MBR, membrane bioreactor; GAC, granular activated carbon; HSSF CW, horizontal subsurface-flow constructed wetland; HRT, hydraulic retention time; LMH, L m^−2^ h^−1^.

b TRL values are provisional assignments by the review authors based on demonstrated environment and scale; the source papers did not report TRLs. Values marked calc. were calculated from source-reported operating parameters and are not measured full-process values.

A simplified techno-economic analysis can be based on the functional unit of 1 m^3^ of wastewater treated to achieve at least 95% removal of parent DCF. The levelized cost of treatment (LCOT) is calculated as:LCOT = [(CAPEX × CRF) + annual OPEX]/annual treated volumewhere CRF = *i*(1 + *i*)*n*/[(1 + *i*)*n* − 1], *i* is the discount rate and *n* is the project lifetime. CAPEX should include oxidation or adsorption reactors, ozone generation and oxygen supply, UV equipment, membranes or activated-carbon contactors, pumps, civil works, monitoring instruments, and automation systems. OPEX should include electricity, oxidants, membrane and UV-lamp replacement, activated-carbon regeneration or replacement, cleaning chemicals, maintenance, analytical monitoring, labour and residual disposal. The principal cost categories, assumptions, and resulting estimates are summarized in Table 7.

**Table 7:** Simplified TEA framework and illustrative cost assessment for achieving ≥95% DCF removal

Cost category	Principal cost drivers
CAPEX	Ozone generator, oxygen supply, contactor, UV reactor and lamps, membrane or carbon contactors, pumps, filtration, civil works, monitoring equipment, PLC/SCADA and contingency
OPEX	Electricity, ozone or H_2_O_2_, activated carbon, membrane and lamp replacement, cleaning chemicals, maintenance, labour, LC-MS/MS verification and residual disposal
Main sensitivities	Plant capacity, utilization, DOC and nitrite, ozone/UV dose, electricity price, membrane lifetime, carbon regeneration frequency and existing filtration
Illustrative result	€0.41 m^−3^ total hybrid cost, €0.36 m^−3^ conventional reference and approximately €0.05 m^−3^ incremental upgrade cost

As an illustrative scenario, Echevarría *et al.* reported >99% DCF removal using ozonation-UV at 6 mg O_3_ L^−1^ and 400 J m^−2^, with an OPEX of €0.158 m^−3^ and a CAPEX of €655 per m^3^ day^−1^ of installed capacity.^180^ After applying a 30% price adjustment and assuming a 20 year lifetime, 5% discount rate and 90% utilization, the estimated levelized cost is approximately €0.41 m^−3^, compared with €0.36 m^−3^ for the conventional ref. 181. Thus, the incremental cost of achieving the DCF target is approximately €0.05 m^−3^ for this very large-scale retrofit. However, contemporary full-scale estimates indicate approximately €0.10–0.20 m^−3^ for quaternary-treatment upgrades at smaller capacities. These values are scenario-specific and depend strongly on plant scale, dissolved organic carbon, oxidant dose, electricity price and existing infrastructure. Moreover, 95% removal of parent DCF does not necessarily imply complete mineralization or elimination of transformation-product toxicity.

Based on the barriers identified in this review, future research should be prioritized according to its capacity to reduce energy and cost while providing reliable evidence of environmental and regulatory compliance. Four high-leverage research priorities are proposed. These priorities integrate fundamental material and biological research with the applied requirements of long-term operation, process control, environmental assessment and investment decision-making. The priority is to standardize performance validation and integrated LCA-TEA protocols. Current cost estimates remain strongly dependent on plant scale, local conditions, system boundaries and operating assumptions.^182^ A minimum reporting framework should include the treatment of 1 m^3^ of real wastewater as the functional unit, parent DCF removal, mineralization, transformation-product toxicity, energy and chemical consumption, media lifetime, residual management, CAPEX, OPEX and uncertainty analysis. Multi-season pilot or full-scale data are particularly important for converting laboratory performance into reliable investment and compliance evidence. The second priority is the development of affordable online sensors and adaptive control systems. UV254 measurements have been successfully used as surrogate parameters for monitoring micropollutant removal and controlling PAC dosage.^183^ However, surrogate measurements cannot precisely quantify every micropollutant. Therefore, future systems should combine online UV254, DOC, ozone residual and operational sensors with periodic DCF and transformation-product verification by LC-MS/MS. Such integration would limit chemical and energy overconsumption while maintaining the required removal performance.

The third priority concerns low-fouling, selective and regenerable membranes and sorbents. Membrane performance is governed by the interactions among membrane properties, operating conditions, wastewater constituents and target contaminants.^184^ Research should consequently focus on fouling-resistant surfaces, selective transport or adsorption mechanisms, low-pressure operation, mild regeneration and long-term stability in real wastewater. These advances would directly reduce pumping energy, cleaning chemicals, replacement frequency and operational downtime. The fourth priority is transformation-product-targeted biological polishing. Full-scale and laboratory-to-full-scale investigations have demonstrated that several ozonation transformation products can remain stable during conventional biological or sand filtration post-treatment.^185,186^ In all, research should identify the metabolic pathways and microorganisms responsible for their degradation and develop stable microbial consortia, biofilms, or enzyme-based systems. Their evaluation must include transformation-product disappearance, mineralization, ecotoxicity and operational resilience. Addressing these four priorities together would provide the technical reliability, economic predictability and compliance evidence required for commercial deployment.

### 6.4. Technical limits of circularity and resource recovery

The integration of circular-economy principles into hybrid DCF treatment should extend beyond high removal efficiency and consider whether materials, energy, and water are genuinely recovered without transferring DCF or its transformation products to another waste stream. The principal circularity opportunities include adsorbent regeneration, energy and material recovery from sludge, treated-water reuse, and reduced consumption of virgin adsorbents and chemicals. However, these benefits must be evaluated against regeneration energy, chemical consumption, loss of adsorption capacity, treatment of concentrated residuals, and final disposal requirements. Adsorbent regeneration can reduce virgin-material consumption, but its technical and economic feasibility is highly material-specific. For example, thermal regeneration of thermo-plasma expanded graphite at 105 °C for 2 h followed by 200 °C for 4 h maintained approximately 100% of its DCF adsorption capacity over four cycles. In contrast, regeneration using 0.2 M NaOH reduced the relative adsorption capacity to approximately 50% after the first cycle and 25% after the second cycle.^187^ Although thermal regeneration was technically more effective, its electricity or fuel demand and the fate of desorbed or thermally transformed DCF must be considered. Chemical regeneration operates at lower temperatures but consumes reagents, produces contaminated washing solutions, and may cause adsorbent losses. Consequently, regeneration should only be considered environmentally and economically advantageous when the avoided production and transport of virgin adsorbent exceed the cumulative energy, chemical, residual-treatment, and capacity-loss burdens over successive cycles.

Hybrid biological processes also offer opportunities for sludge valorization through anaerobic digestion and the recovery of biogas, heat, nutrients, or volatile fatty acids.^188,189^ Nevertheless, energy recovery does not necessarily demonstrate complete DCF removal. During anaerobic fermentation of waste-activated sludge, only approximately 28.8% and 45.8% of DCF were degraded under batch and long-term operating conditions, respectively. Therefore, digestate, liquid sidestreams, and transformation products require targeted monitoring and, where necessary, additional polishing treatment before nutrient recovery, land application, or water reuse can be considered safe. The recovery of DCF itself from membrane concentrates, adsorbent regenerants, or DCF-containing brines is generally unlikely to have practical value because these streams contain complex mixtures of organic matter, salts, co-contaminants, and transformation products. Unless a sufficiently concentrated and pure product with a legitimate reuse pathway can be demonstrated, these streams should be classified as secondary wastes requiring destructive treatment. Similarly, AOP-generated intermediates should not be described as valuable by-products. Ozonation studies have demonstrated that numerous transformation products can remain after oxidation and may persist through conventional post-treatment, confirming the need for activated-carbon, biological, or other polishing steps.^186^

Accordingly, the most realistic circular strategy is a hierarchical one: first, minimize energy and chemical inputs; second, extend adsorbent and membrane lifetimes; third, recover useful energy, nutrients, and treat water where safety criteria are satisfied; and finally, destructively treat DCF-rich concentrates and non-recoverable residuals. Circularity claims should therefore be supported by mass balances, toxicity testing, regeneration-cycle data, and combined LCA and TEA rather than by DCF removal efficiency alone.

## 7. Conclusions and future perspectives

This review shows that hybridization is advantageous only when the unit processes perform complementary functions – load reduction or concentration, separation, transformation, and polishing – and when that complementarity produces a measurable operational or environmental benefit. No configuration is universally optimal. For municipal secondary effluents containing dilute DCF, adsorption-membrane systems currently offer one of the most credible near-term polishing options: continuous PAC/UF studies in real municipal effluent reported approximately 83–85% DCF removal, and the pressurized configuration operated at 0.056 kWh m^−3^. This combination can reduce dissolved DCF and organic foulants while maintaining membrane productivity, but it transfers contaminant mass to PAC and residual streams and does not, by itself, demonstrate attainment of ng L^−1^ receiving-water targets. For municipal or hospital wastewater with a high biodegradable organic load, biological-AOP sequences are more rational because biological treatment first removes bulk organic matter, thereby lowering radical scavenging and oxidant demand before oxidative polishing. When toxicity or recalcitrance limits biological treatment, the reverse AOP-biological sequence can increase biodegradability, provided that the resulting transformation products are shown to be biologically removed. For water reuse or treatment of membrane concentrates, membrane-AOP systems can couple high-quality separation with destruction of the concentrated residual, although fouling, membrane oxidation, energy use, and concentrate management remain decisive constraints. Adsorption-AOP materials, MOF-based self-cleaning membranes, plasma hybrids, and CO_2_-assisted systems show strong laboratory potential, but the available evidence is not yet sufficient to classify them as market-ready for continuous operation in complex wastewater. Across these application scenarios, the principal limitation of the evidence base is not the absence of high removal percentages, but the scarcity of comparable long-term data under realistic conditions. Values above 90% are frequently obtained in synthetic water, batch experiments, or at DCF concentrations far above those encountered in municipal effluents. Natural organic matter, inorganic ions, surfactants, co-occurring pharmaceuticals, and variable hydraulic loads can reduce adsorption, scavenge reactive species, accelerate membrane fouling, and inhibit or restructure microbial communities. Moreover, percentage removal alone cannot establish compliance with a 4 or 40 ng L^−1^ receiving-water benchmark; influent and effluent concentrations, method quantification limits, and temporal variability must also be reported. A decline in parent DCF likewise cannot be equated with detoxification or mineralization, because persistent or more toxic transformation products can accumulate, while adsorption, membrane separation, and sludge production may merely transfer DCF to spent solids, retentates, regeneration eluates, or sludge. Consequently, a hybrid process should be considered commercially and environmentally credible only when removal, destruction, residual toxicity, energy and chemical demand, cost, and final management of all secondary streams are evaluated together. Regeneration should be described as circular only when repeated-cycle performance and a life-cycle comparison demonstrate lower virgin-material demand and lower overall burden, with the desorbed or concentrated DCF ultimately destroyed or safely managed.

Future research should therefore proceed in a prioritized sequence. First, the highest-leverage need is long-term, continuous pilot- and full-scale validation in real municipal, hospital, and pharmaceutical wastewater across seasonal and loading variability, using analytical methods that can verify the required final concentrations. Second, studies should close the DCF mass balance across water, biomass or sludge, adsorbent, membrane concentrate, and regeneration streams, while combining target and non-target transformation-product analysis with TOC and ecotoxicity measurements; this is essential to distinguish true destruction from phase transfer. Third, standardized reporting and harmonized TEA/LCA protocols are required, including *C*_0_ and *C*_f_, quantification limits, HRT or EBCT, throughput, membrane flux and recovery, fouling and cleaning frequency, specific energy or electrical energy per order, reagent consumption, regeneration cycles, residual generation, CAPEX, OPEX, and clearly defined LCA functional units and system boundaries. Fourth, materials research should focus on low-fouling membranes, non-leaching and reusable catalysts, regenerable adsorbents, and resilient biological polishing communities that remove persistent transformation products, with durability demonstrated over repeated cycles rather than inferred from short tests. Fifth, online sensing and adaptive control should be evaluated against well-optimized fixed operation: site-calibrated UV254 or fluorescence surrogates and process measurements may support control, but they require periodic LC-MS/MS confirmation and evidence of lower life-cycle cost or greater robustness. Near-term deployment should therefore favor modular, fit-for-purpose retrofits selected from the wastewater matrix, treatment target, available infrastructure, energy supply, and capacity to manage residuals. Point-source treatment can reduce concentrated hospital or industrial loads, whereas centralized polishing remains necessary for diffuse municipal inputs; the two approaches should be viewed as complementary barriers. This evidence-based selection framework, rather than maximum laboratory removal alone, is the most credible pathway for translating hybrid DCF treatment from promising experiments to reliable and sustainable practice.

## Author contributions

Rodrigo Gutiérrez-Ramírez: investigation, data curation, writing – original draft. Samira El Omari: investigation, data curation, writing – original draft. Mehdi Mennani: investigation, data curation, writing – original draft. Leila Azaryoh: investigation, data curation, writing – original draft. Mohamed Laabd: investigation, data curation, writing – original draft. Zineb Kassab: investigation, data curation, writing – original draft. Fabián Férnandez-Luqueño: supervision. Youness Abdellaoui: conceptualization, supervision, validation, writing – review & editing.

## Conflicts of interest

There are no conflicts to declare.

## Supplementary Material

RA-016-D6RA05914G-s001

## Data Availability

The data used for the literature-based analysis were obtained from previously published studies, all of which are cited in the manuscript and supplementary information (SI). Supplementary information is available. See DOI: https://doi.org/10.1039/d6ra05914g.
