# Supplementary material for: Sustainable hybrid technologies for diclofenac disposal: from innovation to commercial feasibility
Source: RSC Adv. 2026 Sep 11;16(43):52011–44. doi: 10.1039/d6ra05914g (PMC13568930; doi:10.1039/d6ra05914g)
Supplement: Supplementary file 1 [file RA-016-D6RA05914G-s001.pdf]

Supplementary Information (SI) for RSC Advances.
This journal is © The Royal Society of Chemistry 2026

Sustainable Hybrid Technologies for Diclofenac Disposal: From Innovation to Commercial Feasibility

Rodrigo GUTIÉRREZ-RAMÍREZ [1], Samira El OMARI [2], Mehdi MENNANI [3], Leila AZARYOUH [3,4], Mohamed LAABD [2], Zineb KASSAB [3], Fabián FERNÁNDEZ-LUQUEÑO [1], Youness ABDELLAOUI [1,5,*]

[1] Cinvestav Saltillo, Sustainability of Natural Resources and Energy, Av. Industria Metalúrgica 1062, Parque Industrial Ramos Arizpe. Ramos Arizpe, Coahuila C.P. 25900, Mexico

[2] Laboratory of Materials and Environment, Faculty of Sciences, Ibn Zohr University, Agadir, Morocco

[3] College of Chemical Sciences and Engineering (CCSE), Department of Materials Science, Energy and Nano-engineering (MSN), Mohammed VI Polytechnic University (UM6P), 43150 Benguerir, Morocco.

[4] Equipe des Procédés Chimiques et Matériaux Appliqués (EPCMA), Faculté Polydisciplinaire de Béni-Mellal, Université Sultan Moulay Slimane, BP 592, 23000 Béni-Mellal, Morocco.

[5] SECIHTI-Cinvestav Saltillo. Environmental Functional Materials Laboratory, Sustainability of Natural Resources and Energy, Ramos Arizpe, Coahuila, Mexico.

The corresponding author:** Prof. Youness Abdellaoui; ***e-mails: abdellaoui.youness@gmail.com; youness@cinvestav.mx

Supplementary Materials

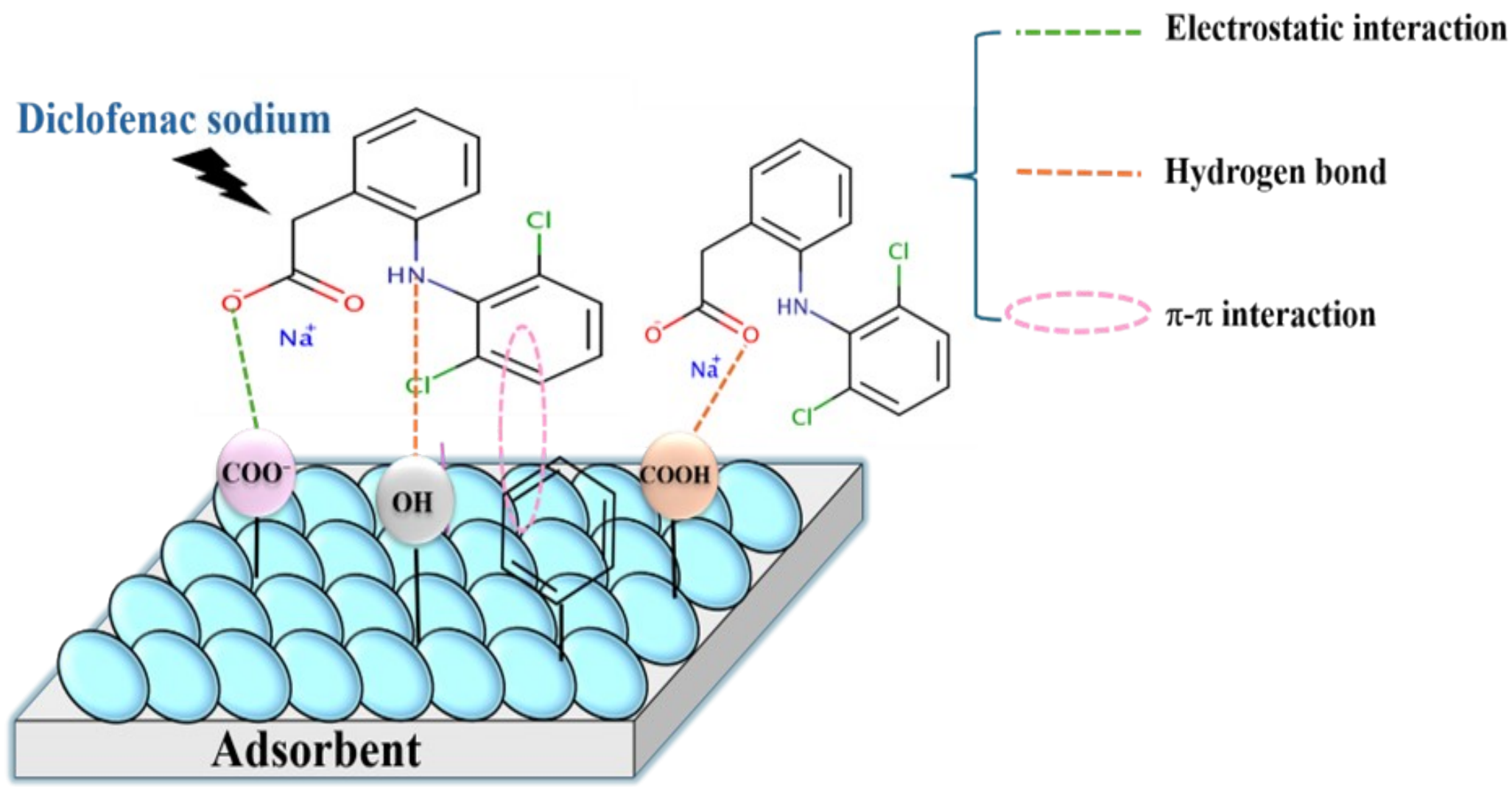

Figure S1. Possible adsorption pathways for DCF.

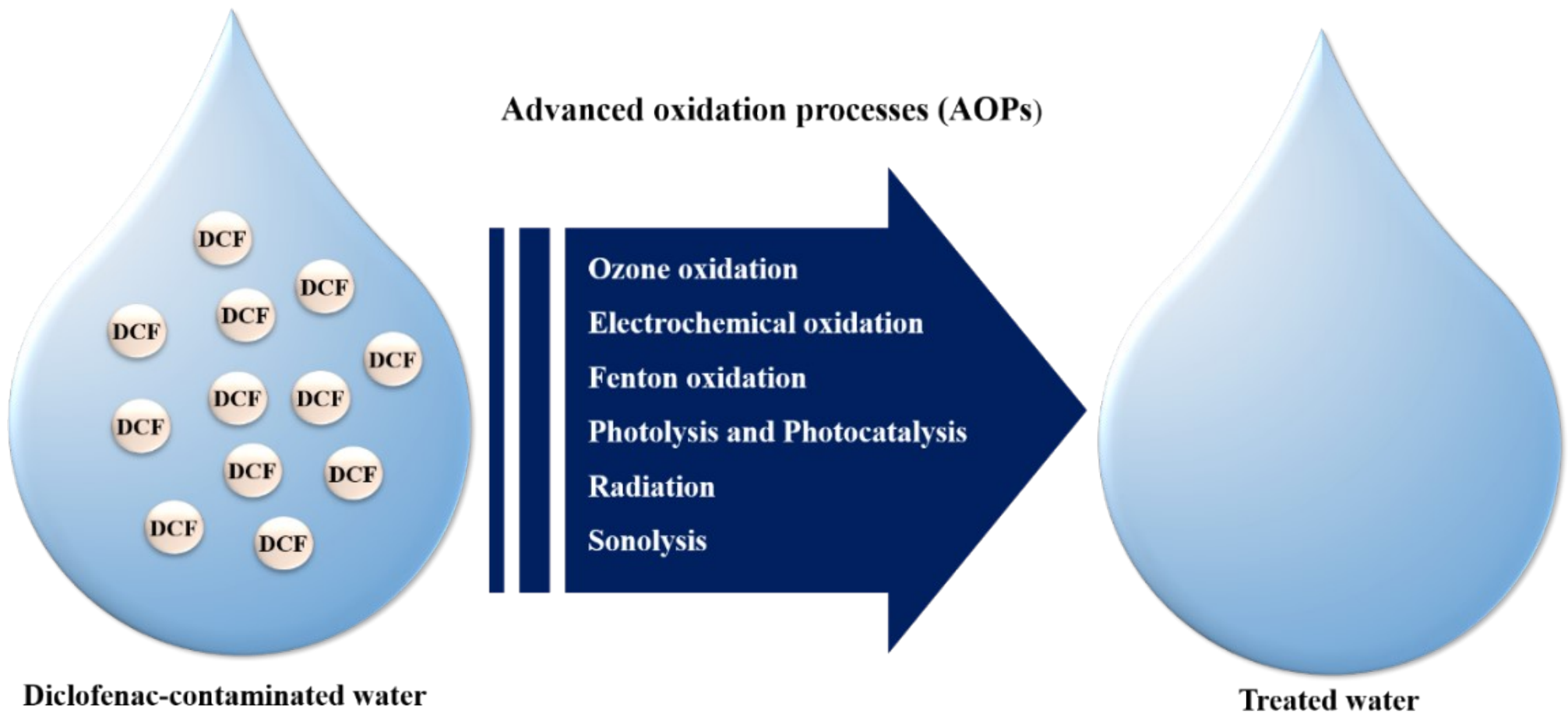

Figure S2. Oxidation processes for treating DCF-contaminated water.

Table S1. Physicochemical characteristics of diclofenac sodium and potassium. Adapted with permission from [1].

	Diclofenac sodium	Diclofenac potassium
Formula	$C_{14}H_{10}Cl_2NNaO_2$	$C_{14}H_{10}Cl_2KNO_2$
Structure	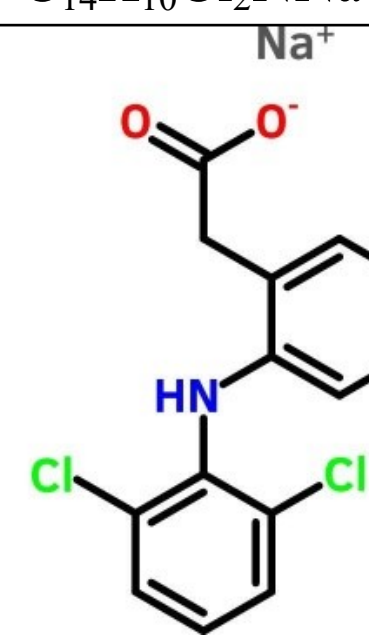	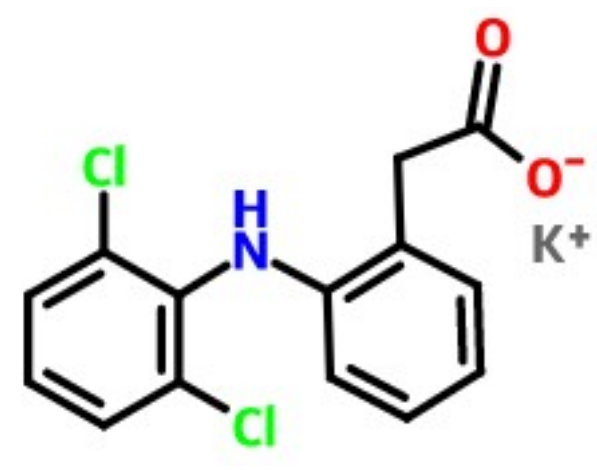
Therapeutic Class	Non-steroidal anti-inflammatory agent	Non-steroidal anti-inflammatory agent
Molecular Mass (g mol^{-1})	318.1	334.2
log K_{ow}	4.51	4.28
pKa	4.15	4.10
Water solubility (mg mL^{-1})	2.37	2.34

Table S2. Recent works using different materials for adsorption of DCF.

Adsorbent material	Adsorption capacity ($mg \cdot g^{-1}$)	Operating conditions			Reference
		pH	Equilibrium time (min)	Adsorbent dosage ($mg \cdot L^{-1}$)	
Calcined kaolinite clay	238	4	120	20	[2]
Ti-pillared montmorillonite clay	23.83	7	60	100	[3]
Glutaraldehyde-modified silica/chitosan composites	237.8	6.7	120	1000	[4]
Magnetic molecularly imprinted polymer (MMIP)	139.7	3-7	200	20	[5]
L@M-MWCNTs	892.85	5	60	1600	[6]
MWCNT@OH-COOH-Fe	168.76	-	-	1.5	[7]
LDH-PmPD	588	4	2	-	[8]
TG-IL hydrogels	327,87	5	360	600	[9]
AmCS@Fe3O4	469.48	4.5	60	400	[10]
Ligand-Doped Silver Triazolate Metal-organic	91-109	10.00	90	1000	[11]

Table S3. Membrane filtration for DCF removal.

Filtration type	Membrane type	Mechanism	Removal efficiency (%)	Reference
Nanofiltration	Desal HL nanofiltration membrane	Physical sieving, electrostatic repulsion, and steric hindrance.	> 90	[12]
Nanofiltration	Loose NF50 nanofiltration membrane	Electrostatic repulsion was the primary mechanism. Charge exclusion also contributed	99.7	[13]
Nanofiltration	Poly(vinyl alcohol), citric acid, glycerol, and green synthesized silver nanoparticles	Steric Effect, Donnan effect and adsorption	80	[14]
Nanofiltration	Piperazine/TMC-based membrane (PIP-KRO)	Size exclusion, electrostatic repulsion, and hydrophobic/hydrophilic interactions	99.5	[15]
Reverse osmosis	RO4	Size exclusion, electrostatic repulsion, and hydrophobic/hydrophilic interactions	99.5	[15]
Reverse osmosis	Filmtec BW30	Electrostatic repulsion, membrane hydrophobicity	99	[16]

Table S4. Performance of biological treatments for DCF.

Type of biological process	System	Removal efficiency (%)	Reference
Co-culture	Microbial consortium (*P. oxalicum XD-3.1, P. rastrickii, and C. cladosporoides)*	>80	[17]
Biodegradation	Bacterial Strains (*Bacillus subtilis* et *Brevibacillus laterosporus)*	100	[18]
Biotransformation	Enhanced Biological Phosphorus Removal (EBPR)	>98	[19]
Activated Sludge	Aerobic	>99% (at high concentration)	[20]
Biodegradation	Indigenous Microflora	70-90	[21]
Biological oxidation	Bio-electrochemical system (BES)	Moderate	[22]
Electrochemical degradation	(BES)	Moderate	
Adsorption	Electrodes	Low to moderate	
Enzyme immobilization	Perlite-HRP	100	[23]
Enzyme immobilization	Laccase	70-80	
Degradation	Microalgae	74.68	[24]

References

1 I. Alessandretti, C. V. T. Rigueto, M. T. Nazari, M. Rosseto and A. Dettmer, *Elsevier Ltd*, 2021, preprint, DOI: 10.1016/j.jece.2021.106743.

2 F. Mansouri, K. Chouchene, A. Wali, J. Labille, N. Roche and M. Ksibi, *Chemosphere*, DOI:10.1016/j.chemosphere.2024.141469.

3 M. Chauhan, V. K. Saini and S. Suthar, *J. Hazard. Mater.*, DOI:10.1016/j.jhazmat.2020.122832.

4 T. S. Machado, L. Crestani, G. Marchezi, F. Melara, J. R. de Mello, G. L. Dotto and J. S. Piccin, *Carbohydr. Polym.*, DOI:10.1016/j.carbpol.2021.118868.

5 H. A. Dakhly, S. A. H. Albohy, A. A. Salman and A. S. Abo Dena, *RSC Adv.*, 2024, **14**, 15942–15952.

6 R. A. Grmasha, O. J. Al-sareji, M. Meiczinger, C. Stenger-Kovács, R. A. Al-Juboori, M. Jakab, E. Lengyel, V. Somogyi, M. A. Khan and K. S. Hashim, *Environ. Res.*, DOI:10.1016/j.envres.2024.118097.

7 H. P. de S. Costa, E. D. V. Duarte, M. G. C. da Silva and M. G. A. Vieira, *Journal of Water Process Engineering*, DOI:10.1016/j.jwpe.2024.104923.

8 T. Xiong, X. Yuan, H. Wang, Z. Wu, L. Jiang, L. Leng, K. Xi, X. Cao and G. Zeng, *Chemical Engineering Journal*, 2019, **366**, 83–91.

9 J. Du, K. Xu, X. Yang, Z. Dong and L. Zhao, *Int. J. Biol. Macromol.*, DOI:10.1016/j.ijbiomac.2024.130758.

10 X. X. Liang, A. M. Omer, Z. hong Hu, Y. guang Wang, D. Yu and X. kun Ouyang, *Chemosphere*, 2019, **217**, 270–278.

11 H. Miao, H. Hou, W. Zhang and G. Yang, *ChemistrySelect*, 2020, **5**, 11948–11954.

12 X. mao Wang, B. Li, T. Zhang and X. yan Li, *Desalination*, 2015, **370**, 7–16.

13 B. Maryam, V. Buscio, S. U. Odabasi and H. Buyukgungor, *Environ. Technol. Innov.*, DOI:10.1016/j.eti.2020.100641.

14 C. Suliani Raota, J. Duarte, J. da Silva Crespo, C. Baldasso and M. Giovanela, *J. Environ. Chem. Eng.*, DOI:10.1016/j.jece.2024.113237.

15 E. Alonso, C. Sanchez-Huerta, Z. Ali, Y. Wang, L. Fortunato and I. Pinnau, *J. Memb. Sci.*, DOI:10.1016/j.memsci.2023.122357.

16 E. B. Tasdemir, M. Pardon, S. Rezaei Hosseinabadi, L. A. J. Rutgeerts, D. Cabooter and I. F. J. Vankelecom, *Membranes (Basel).*, DOI:10.3390/membranes15060183.

17 G. Angeles-de Paz, A. Ledezma-Villanueva, T. Robledo-Mahón, C. Pozo, C. Calvo, E. Aranda and J. Purswani, *Chemosphere*, DOI:10.1016/j.chemosphere.2022.137472.

18 C. Grandclément, A. Piram, M. E. Petit, I. Seyssiecq, I. Laffont-Schwob, G. Vanot, N. Tiliacos, N. Roche and P. Doumenq, *J. Chem.*, DOI:10.1155/2020/9789420.

19 S. Kolakovic, R. Salgado, E. B. Freitas, M. R. Bronze, M. T. Sekulic, G. Carvalho, M. A. M. Reis and A. Oehmen, *Science of the Total Environment*, DOI:10.1016/j.scitotenv.2021.151232.

20 M. S. Elshikh, D. S. Hussein, F. S. Al-khattaf, R. A. Rasheed El-Naggar and K. S. Almaary, *Environ. Res.*, DOI:10.1016/j.envres.2022.112723.

21 M. Matejczyk, P. Ofman, K. Dąbrowska, R. Świsłocka and W. Lewandowski, *J. Environ. Sci. (China)*, 2020, **91**, 128–141.

22 B. A. J. Poursat, F. Rempe, J. Pereira, N. B. Sutton and A. ter Heijne, *Science of the Total Environment*, DOI:10.1016/j.scitotenv.2024.173932.

23 A. Vardanyan, T. Agback, O. Golovko, Q. Diétre and G. A. Seisenbaeva, *ACS ES and T Water*, 2024, **4**, 751–760.

24 F. Bano, A. Malik and S. Z. Ahammad, *Sustainability (Switzerland)*, DOI:10.3390/su13147690.
